# IFNAR1-Signalling Obstructs ICOS-mediated Humoral Immunity during Non-lethal Blood-Stage *Plasmodium* Infection

**DOI:** 10.1371/journal.ppat.1005999

**Published:** 2016-11-03

**Authors:** Ismail Sebina, Kylie R. James, Megan S. F. Soon, Lily G. Fogg, Shannon E. Best, Fabian de Labastida Rivera, Marcela Montes de Oca, Fiona H. Amante, Bryce S. Thomas, Lynette Beattie, Fernando Souza-Fonseca-Guimaraes, Mark J. Smyth, Paul J. Hertzog, Geoffrey R. Hill, Andreas Hutloff, Christian R. Engwerda, Ashraful Haque

**Affiliations:** 1 Malaria Immunology Laboratory, QIMR Berghofer Medical Research Institute, Herston, Queensland, Australia; 2 The University of Queensland, School of Medicine PhD Program, Herston, Queensland, Australia; 3 Immunology and Infection Laboratory, QIMR Berghofer Medical Research Institute, Herston, Queensland, Australia; 4 Immunity in Cancer and Infection Laboratory, QIMR Berghofer Medical Research Institute Herston, Queensland, Australia; 5 Hudson Institute of Medical Research, Clayton, Victoria, Australia; 6 Bone Marrow Transplantation Laboratory, QIMR Berghofer Medical Research Institute, Herston, Queensland, Australia; 7 Chronic Immune Reactions, German Rheumatism Research Centre (DRFZ), a Leibniz Institute, Berlin, Germany; Faculdade de Medicina da Universidade de Lisboa, PORTUGAL

## Abstract

Parasite-specific antibodies protect against blood-stage *Plasmodium* infection. However, in malaria-endemic regions, it takes many months for naturally-exposed individuals to develop robust humoral immunity. Explanations for this have focused on antigenic variation by *Plasmodium*, but have considered less whether host production of parasite-specific antibody is sub-optimal. In particular, it is unclear whether host immune factors might limit antibody responses. Here, we explored the effect of Type I Interferon signalling via IFNAR1 on CD4^+^ T-cell and B-cell responses in two non-lethal murine models of malaria, *P*. *chabaudi chabaudi* AS (*Pc*AS) and *P*. *yoelii* 17XNL (*Py*17XNL) infection. Firstly, we demonstrated that CD4^+^ T-cells and ICOS-signalling were crucial for generating germinal centre (GC) B-cells, plasmablasts and parasite-specific antibodies, and likewise that T follicular helper (Tfh) cell responses relied on B cells. Next, we found that IFNAR1-signalling impeded the resolution of non-lethal blood-stage infection, which was associated with impaired production of parasite-specific IgM and several IgG sub-classes. Consistent with this, GC B-cell formation, Ig-class switching, plasmablast and Tfh differentiation were all impaired by IFNAR1-signalling. IFNAR1-signalling proceeded via conventional dendritic cells, and acted early by limiting activation, proliferation and ICOS expression by CD4^+^ T-cells, by restricting the localization of activated CD4^+^ T-cells adjacent to and within B-cell areas of the spleen, and by simultaneously suppressing Th1 and Tfh responses. Finally, IFNAR1-deficiency accelerated humoral immune responses and parasite control by boosting ICOS-signalling. Thus, we provide evidence of a host innate cytokine response that impedes the onset of humoral immunity during experimental malaria.

## Author Summary

*Plasmodium* parasites cause malaria by invading, replicating within, and rupturing out of red blood cells. Natural immunity to malaria, which depends on generating *Plasmodium*-specific antibodies, often takes years to develop. Explanations for this focus on antigenic variation by the parasite, but consider less whether antibody responses themselves may be sub-optimal. Surprisingly little is known about how *Plasmodium*-specific antibody responses are generated in the host, and whether these can be enhanced. Using mouse models, we found that cytokine-signalling via the receptor IFNAR1 delayed the production of *Plasmodium-*specific antibody responses. IFNAR1-signalling hindered the resolution of infection, and acted early via conventional dendritic cells to restrict CD4^+^ T-cell activation and their interactions with B-cells. Thus, we reveal that an innate cytokine response, which occurs during blood-stage *Plasmodium* infection in humans, obstructs the onset of antibody–mediated immunity during experimental malaria.

## Introduction

Although robust immunity to malaria is difficult to generate in humans through natural infection or vaccination [1,2], it is nonetheless clear that *Plasmodium*-specific antibodies offer the best known form of immunological protection against blood-stage parasites [3,4,5,6,7,8], and may also control liver-infective sporozoites [9,10]. Considering that a highly effective malaria vaccine remains elusive, it is important to understand how the onset of humoral immunity to blood-stage *Plasmodium* parasites is controlled, and whether this process can be boosted, to accelerate or otherwise enhance antibody-mediated immunity to malaria.

Mouse models of resolving, non-lethal blood-stage *Plasmodium* infection are useful for studying humoral immunity to malaria, since mice fail to control parasitemias and display increased disease severity in the absence of parasite-specific antibodies [4,11,12,13,14]. However, our understanding of how humoral immune responses develop in these models is currently modest. CD4^+^ T follicular helper (Tfh) cells and their associated cytokines, such as IL-21, and germinal centre (GC) B-cells are critical mediators of humoral immune responses in many systems [15,16], and appear to be similarly important during experimental malaria. For instance, an anti-parasitic role for T-cell-derived IL-21 was recently described during non-lethal *Plasmodium chabaudi chabaudi* AS (*Pc*AS) infection [6]. Other recent studies using non-lethal *Plasmodium yoelii* 17XNL (*Py*17XNL) infection focused on co-stimulatory markers on CD4^+^ T-cells, and demonstrated that Programmed cell Death 1 (PD-1) and LAG-3 blockade, or stimulation via OX40 boosted Tfh and GC B-cell responses, with positive effects on parasite control [4,17]. With the exception of these reports, *in vivo* studies of Tfh cells and GC B-cells during experimental malaria remain sparse. Moreover, while these recent reports focused on molecules expressed by CD4^+^ T-cells themselves, less effort has been directed towards determining whether T-cell extrinsic factors, such as innate or inflammatory cytokines, can control humoral immunity.

It is becoming increasingly clear that inducible T-cell co-stimulatory (ICOS) receptor on CD4^+^ T-cells is vital for Tfh cell-dependent humoral immunity across numerous model systems [18,19]. ICOS has been implicated in Tfh differentiation via the stabilization of the transcription factor B-cell lymphoma-6 (Bcl-6) [18,20,21]. Importantly, ICOS supports interactions of emerging Tfh cells with ICOS ligand (ICOSL)-expressing bystander B-cells at the periphery of B-cell follicles, a pivotal process for GC B-cell formation and maintenance [22,23]. Moreover, ICOS facilitates the expression of CXCR5, a chemokine receptor essential for Tfh migration into B-cell zones [18,24]. Despite fundamental roles for ICOS on CD4^+^ T-cells in generating and optimizing B-cell responses and antibody production, its role during blood-stage *Plasmodium* infection was largely unexplored until recently [25], when Wikenheiser *et al*. described weaker Tfh and B-cell differentiation in ICOS-deficient mice after the first week of *Pc*AS infection. Furthermore, although T-cell-intrinsic mechanisms have been defined for regulating CD4^+^ T-cell ICOS levels, for example via Roquin1 and 2 [26,27] and microRNA146a [28], whether or not T-cell extrinsic mechanisms can also modulate ICOS is unclear at present.

Type I interferon (IFN-I) signalling can amplify adaptive immune responses [29,30,31], and drive humoral immunity *in vivo*, particularly in the context of immunization [32,33], viral infection [34,35] and autoimmunity [29,36]. Furthermore, IFN-I-signalling was reported to induce Bcl6, CXCR5 and PD1 expression in naïve CD4^+^ T cells following TCR stimulation *in vitro* [37]. IFN-I-related immune responses have also been observed in PBMC from malaria patients [38,39,40]. Although their functional relevance in humans remains to be established, we recently showed in *ex vivo* cultures of PBMC from *P*. *falciparum-*infected humans, that signalling via IFNAR2 was immunoregulatory [41]. In addition, we showed in experimental mice that IFNAR1-signalling, acting via conventional dendritic cells (cDCs) [42], and employing the canonical IFN-I transcription factor, IRF7 but not IRF3 [43], suppressed Th1 responses and parasite control during experimental severe malaria caused by *P*. *berghei* ANKA (*Pb*ANKA). Recent data has suggested that increased Th1 responses might suppress Tfh cells via IFNγ-signalling in experimental malaria [17], and viral infection [44]. Therefore, the current literature supports a model in which IFNAR1/2-signalling suppresses Th1 responses yet promotes Tfh-dependent humoral immunity during blood-stage *Plasmodium* infection. The aim of this paper was to determine the effect of IFNAR1-signalling on humoral immune responses during experimental malaria.

In this report, we investigated roles for CD4^+^ T cells, ICOS- and IFNAR1-signalling pathways in the development of humoral immune responses during blood-stage *Plasmodium* infection. We confirmed crucial roles for CD4^+^ T-cells and ICOS-signalling in controlling B-cell responses and anti-parasitic immunity. We showed that IFNAR1-signalling obstructed parasite control and antibody production, which was associated with regulation of numerous aspects of the humoral immune response including GC B-cell and plasmablast generation. In particular, IFNAR1-signalling acted early to limit proliferation and localization of activated CD4^+^ T-cells adjacent to and within B-cell follicles in the spleen. Finally, IFNAR1-deficiency boosted humoral immune responses and improved parasite control in an ICOS-dependent manner. Thus, we describe here the restrictive effect of an innate cytokine-signalling pathway on antibody-mediated immunity during experimental blood-stage malaria.

## Results

### GC B-cell and plasmablast differentiation requires CD4^+^ T-cells and ICOS-signalling during blood-stage *Plasmodium* infection

**Fig 1:**
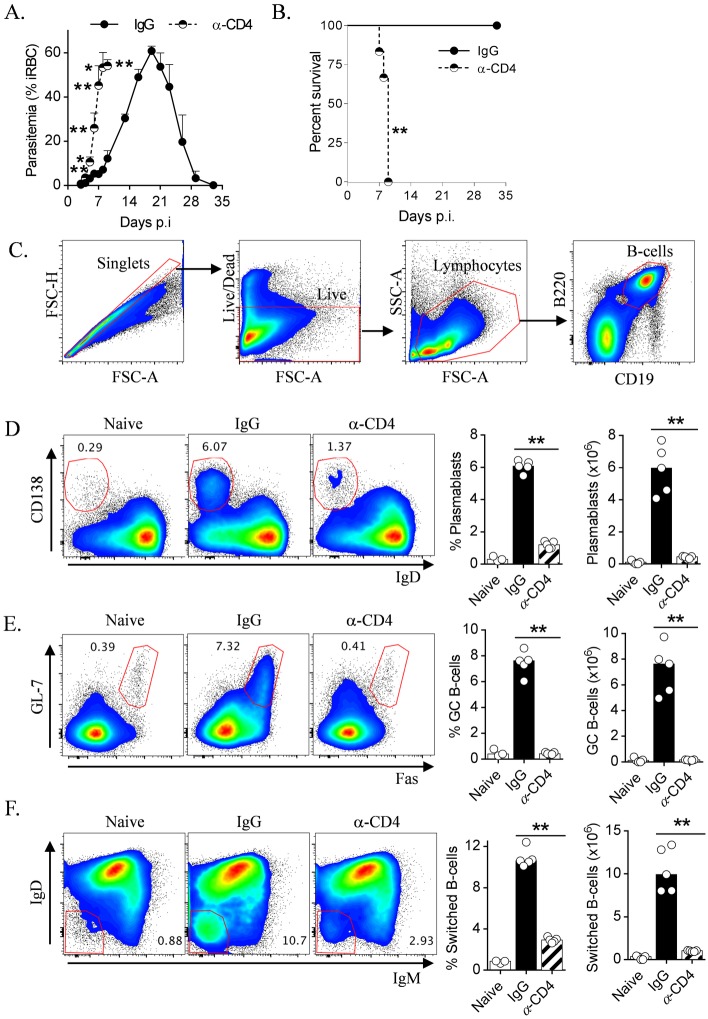
Splenic Germinal Centre B-cell and plasmablast responses are strongly dependent on CD4^+^ cells during blood-stage *Plasmodium* infection. (A) Parasitemia and (B) survival of WT mice (n = 6) treated with CD4-depleting monoclonal antibody (αCD4) or control IgG 1 day prior to infection with *Py*17XNL. (C) Flow cytometric gating strategy employed to analyze splenic B-cell responses throughout the manuscript. (D-F) WT mice (n = 5) were administered αCD4 or control-IgG prior to *Py*17XNL infection. Presented are representative FACS plots (gated on B220^+^ CD19^+^ live singlets), proportions and absolute numbers in the spleen of (D) plasmablasts (IgD^lo^ CD138^hi^), (E) GC B-cells (GL-7^+^ Fas^+^), and (F) Ig-switched B-cells (IgD^lo^ IgM^lo^ cells) from naïve and infected, control IgG and αCD4-treated WT mice, 10 days *p*.*i*. Statistics: Mann-Whitney U test, **P<0.01, except (B) in which Log-rank test applied (** p = 0.0012). Experiment in A&B done once, C-F representative of two independent experiments.

CD4^+^ T-cells are critical for control and resolution of blood-stage *Plasmodium* infection [4,11,45], a phenomenon we first confirmed in *Py*17XNL-infected WT mice depleted of CD4^+^ cells (Fig 1A & 1B). Despite this, to our knowledge there remained no direct evidence that CD4^+^ T cells promoted B-cell responses during experimental malaria. To examine this, wild-type (WT) mice depleted of CD4^+^ cells or given control IgG, were infected with *Py*17XNL and examined for resulting splenic GC B-cell and plasmablast responses (Fig 1C). By day 10 post-infection (*p*.*i*), a timepoint just before the CD4-depleted mice began to succumb to infection, plasmablast differentiation was 80% lower in CD4-depleted mice compared to infected controls (Fig 1D), and GC B-cell formation (Fig 1E) was almost abrogated (95% reduction compared to controls), with a similar, substantial impairment in Ig-class switching (Fig 1F). Together, these data formally demonstrated that GC B-cell and plasmablast generation was highly dependent upon CD4^+^ T-cells.

**Fig 2:**
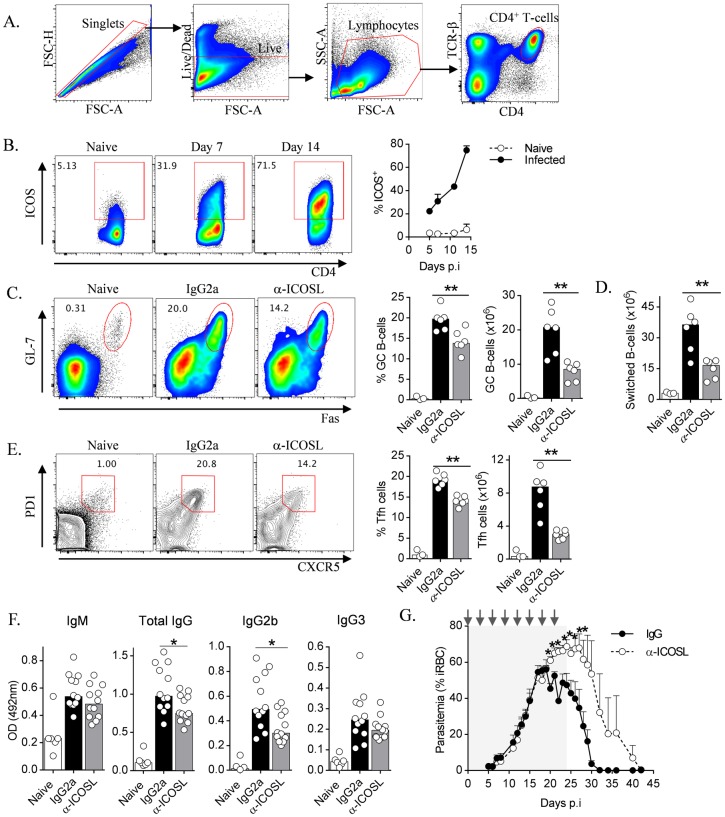
ICOS-signalling promotes humoral immune responses during blood-stage infection. (A) Flow cytometric gating strategy employed to analyze splenic CD4^+^ T-cell responses throughout the manuscript. (B) Representative FACS plots and time-course analysis of cell-surface ICOS expression on splenic CD4^+^ T-cells from WT mice (n = 3/time point) during *Py*17XNL infection. (C-F) WT mice (n = 6/group) were treated with anti-ICOSL blocking monoclonal antibody (α-ICOSL) or its isotype control (rat-IgG2a) prior to and during infection with *Py*17XNL. (C) Representative FACS plots (gated on B220^+^ CD19^+^ live singlets), proportions and numbers of splenic GC B-cells (GL-7^+^ Fas^+^), (D) numbers of splenic Ig-class switched (IgD^lo^ IgM^lo^) B-cells, and (E) representative FACS plots (gated on CD4^+^ TCRβ^+^ live singlets), proportions and numbers of splenic Tfh cells (PD1^+^ CXCR5^+^), in naïve mice and infected, α-ICOSL and control IgG-treated mice, 16 days *p*.*i*. (F) *Py*17XNL-specific IgM, total IgG, IgG2b and IgG3 levels in serum of naïve and infected, α-ICOSL and control IgG-treated mice, 16 days *p*.*i*. (G) Parasitemias in WT mice (n = 6/group) infected with *Py*17XNL, and treated every three days with α-ICOSL or control IgG until day 21 *p*.*i*. (depicted by arrows, with estimated period of cover highlighted with shaded grey box—an α-ICOSL-treated mouse succumbed to infection on each of days 15, 17 and 30 *p*.*i*., and one control-IgG treated mouse succumbed on day 20 *p*.*i*. Data representative of two independent experiments in (B-E), and two pooled independent experiments in (F), with experiment in (G) being conducted once. Statistics: Mann-Whitney U test, *P<0.05; **P<0.01.

While previous studies in mice and humans demonstrated that ICOS expressed on CD4^+^ T- cells was critical for effective humoral responses [18,19,23,46,47], until recently, no such studies had been performed during *Plasmodium* infection [25]. Therefore, we first examined ICOS expression by CD4^+^ T-cells during *Py*17XNL infection (Fig 2A), revealing significant up-regulation by 5–7 days *p*.*i*. (Fig 2B), which progressively increased over the following 7–9 days. To examine a possible functional role for ICOS in the development of humoral immunity, WT mice were treated with α-ICOSL-blocking antibody (α-ICOSL) during *Py*17XNL-infection. Consistent with other experimental models [18,19,48], ICOSL-blockade impaired GC B-cell formation (Fig 2C), Ig-class switching (Fig 2D), Tfh differentiation (Fig 2E), and early production by day 16 *p*.*i*. of parasite-specific total IgG and IgG2b, but not IgM or IgG3 in the serum (Fig 2F). Day 16 *p*.*i*. was an important timepoint during *Py*17XNL infection because it marked the point at which the rate of increase in parasitemia began to slow in WT mice, thus indicating the beginning of the resolution phase of infection. Finally, ICOSL-blockade over the first three weeks of infection exacerbated parasitemias and delayed resolution of infection (Fig 2G). Taken together, these data indicated that ICOS-signalling promoted CD4^+^ T-cell dependent humoral immune responses and parasite control during *Py*17XNL-infection.

### IFNAR1-signalling impairs B-cell and antibody responses during blood-stage *Plasmodium* infection

**Fig 3:**
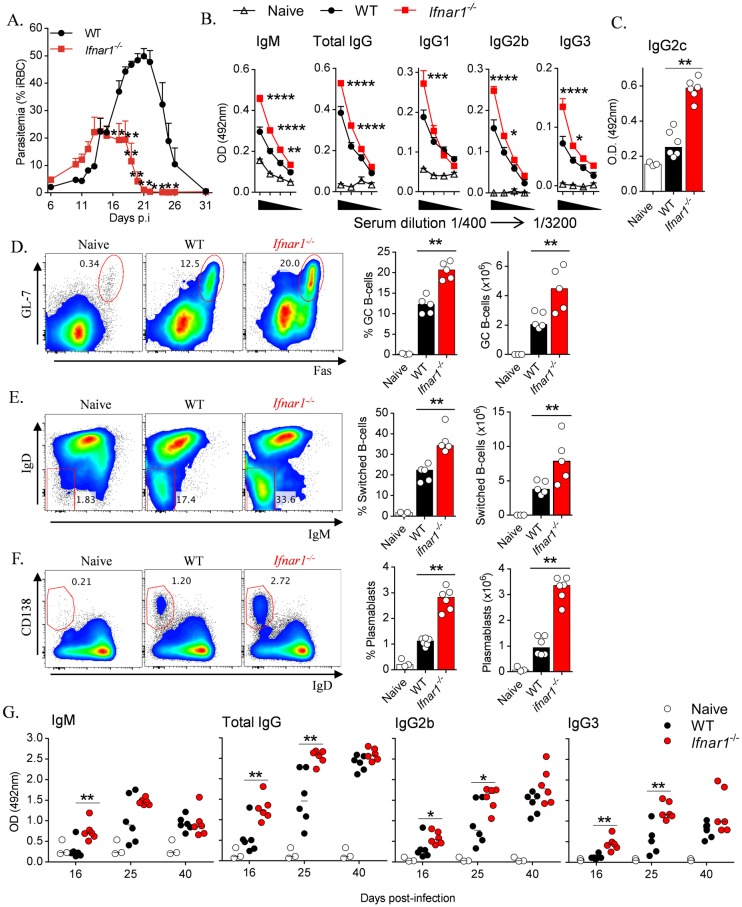
IFNAR1-signalling obstructs B-cell and parasite-specific antibody responses during blood-stage infection. (A) Time-course analysis of parasitemia in WT and *Ifnar1* ^*-/-*^mice (n = 5/group) infected with *Py*17XNL. (B) ELISA quantitation of *Py*17XNL-specific IgM, total IgG, IgG1, IgG2b, IgG3 in serum diluted from 1 in 400 in two-fold sequential dilutions to 1 in 3200, and (C) IgG2c at 1 in 400 in the sera of naïve and infected WT and *Ifnar1* ^*-/-*^ mice, 16 days *p*.*i*. (D&E) Representative FACS plots (gated on B220^+^ CD19^+^ live singlets), proportions and absolute numbers of (D) splenic GC B-cells (GL7^+^ Fas^+^) and (E) Ig-class-switched B-cells (IgD^lo^ IgM^lo^) in naïve and infected WT and *Ifnar1* ^*-/-*^ mice, 16 days *p*.*i*. (F) Representative FACS plots (gated on B220^+^ CD19^+^ live singlets), proportions and absolute numbers of splenic plasmablasts (IgD^lo^CD138^hi^) in naïve and infected WT and *Ifnar1* ^*-/-*^ mice (n = 6), 6 days *p*.*i*. (G) Time course analysis of IgM, total IgG, IgG2b and IgG3 levels in naïve and infected WT and *Ifnar1* ^*-/-*^ mice (n = 6). Statistics: Mann-Whitney U test (A & C-G), Two way ANOVA and Tukey’s test for multiple comparisons in (B), *P<0.05; **P<0.01; ***P<0.001; ****P<0.0001. Data representative of three independent experiments for (A), (D) and (E), two for (B) and (F) and one for (C) and (G).

We next examined the impact of IFNAR1-signalling on parasite control and humoral immune responses during *Py*17XNL-infection. *Ifnar1* ^*-/-*^ mice displayed similar initial parasitemias compared to infected WT controls for the first two weeks of infection, but thereafter exhibited faster control of blood-stage parasites than WT controls (Fig 3A). Similar effects were also observed during *Pc*AS-infection (S1A Fig). Next, we noted increased parasite-specific IgM and total IgG levels in the sera of *Py*17XNL-infected *Ifnar1* ^*-/-*^ mice compared to WT controls at day 16 *p*.*i*. (Fig 3B & 3C). More specifically, levels of parasite-specific IgG1, IgG2b, IgG2c and IgG3 were all higher in *Ifnar1* ^*-/-*^ mice compared to WT controls (Fig 3B & 3C). Next, we noted that GC B-cell (Fig 3D) and Ig-switched B-cell generation (Fig 3E) was limited by IFNAR1-signalling at day 16 *p*.*i*. In addition, IFNAR1-signalling impaired plasmablast formation at day 6 *p*.*i*. (Fig 3F). We also observed similar regulation of plasmablasts and emerging GC B-cells during *Pc*AS-infection (S1B & S1C Fig). Finally, we explored the longer-term effect of IFNAR1-signalling on parasite-specific antibody production. At day 25 *p*.*i*. with *Py*17XNL, *Ifnar1* ^*-/-*^ mice maintained higher serum IgG levels, including IgG2b and IgG3, but not IgM, compared to WT controls (Fig 3G). By day 40 *p*.*i*., total IgG, IgG2b and IgG3 concentrations had risen further to similar levels in WT and *Ifnar1* ^*-/-*^ mice, while IgM levels had dropped, again with no differences between groups (Fig 3G). Taken together, our data indicated that IFNAR1-signalling delayed parasite control, B-cell responses and the onset of antibody production during blood-stage *Plasmodium* infection.

### IFNAR1-signalling limits Tfh differentiation and CD4^+^ T cell localization adjacent to B-cell areas of the spleen

**Fig 4:**
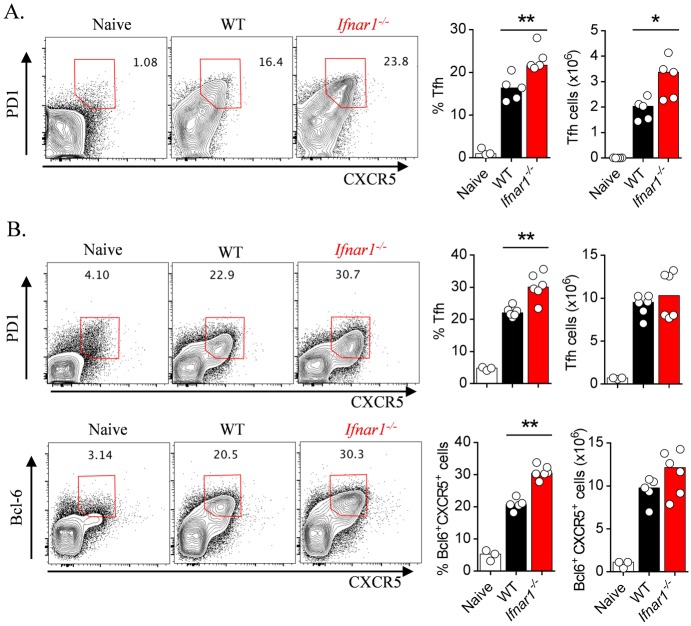
IFNAR1-signalling limits splenic Tfh cell responses. (A) WT and *Ifnar1* ^*-/-*^ mice (n = 5/group) were infected with *Py*17XNL, and splenic Tfh (PD1^+^ CXCR5^+^) cell proportions and absolute numbers were assessed at day 16 *p*.*i*. Representative FACS plots gated on CD4^+^ TCRβ^+^ live singlets. Data representative of three independent experiments; Mann-Whitney U test *P<0.05; **P<0.01. (B) WT *and Ifnar1* ^*-/-*^ mice (n = 5-6/group) were infected with *Pc*AS, and splenic Tfh (PD1^+^ CXCR5^+^ or Bcl-6^+^ CXCR5^+^) cell proportions and absolute numbers were assessed at day 8 *p*. *i*. Representative FACS plots gated on CD4^+^ TCRβ^+^ live singlets. Data representative of three independent experiments; Mann-Whitney U test *P<0.05; **P<0.01.

**Fig 5:**
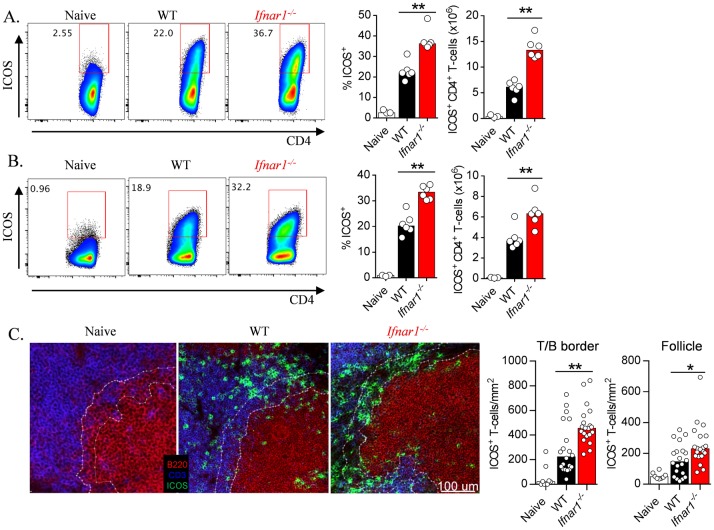
IFNAR1-signalling restricts CD4^+^ T-cell expression of ICOS and proximity to B cell areas of the spleen. (A) Representative FACS plots (gated on CD4^+^ TCRβ^+^ live singlets), proportions and absolute numbers of splenic ICOS^+^ CD4^+^ T-cells in naïve, and *Py*17XNL-infected WT and *Ifnar1* ^*-/-*^mice (n = 6 per group) 6 days *p*.*i*. Experiment performed once. Mann-Whitney U test **P<0.01. B) Representative FACS plots (gated on CD4^+^ TCRβ^+^ live singlets), proportions and absolute numbers of splenic ICOS^+^ CD4^+^ T-cells in naïve, and *Pc*AS-infected WT and *Ifnar1* ^*-/-*^mice (n = 6 per group) 6 days *p*.*i*. Data representative of three independent experiments. Mann-Whitney U test **P<0.01. C) Representative confocal microscopy images showing spleen sections from naïve (n = 2) and *Pc*AS-infected WT and *Ifnar1* ^*-/-*^mice (n = 5) on day 5 *p*.*i*., stained for ICOS (Green), CD3 (Blue), and B220 (Red). T-B borders are outlined by white dotted lines. Summary graphs illustrate ICOS^+^ CD3^+^ cell densities for individual T/B borders and B cell follicles, with data pooled from four T-B borders or follicles per mouse, (n = 2 for naïve and n = 5 for WT and *Ifnar1* ^*-/-*^ mice). Scale bar: 100μm. Mann-Whitney U test *P<0.05; **P<0.01.

Given that GC B-cell and plasmablast formation was dependent on CD4^+^ T cells (Fig 1D–1F), and likewise that Tfh differentiation depended on the presence of B-cells (S2 Fig), we next examined the impact of IFNAR1-signalling on splenic CD4^+^ T-cell responses (Fig 4). Compared to infected WT controls, *Ifnar1* ^*-/-*^ mice displayed increased Tfh proportions and numbers during *Py*17XNL infection (Fig 4A), and increased proportions during *Pc*AS infection (Fig 4B). We chose to assess emerging Tfh cells around day 6–8 *p*.*i*. during *Pc*AS infection, since our previous work had suggested that strong CD4^+^ T helper cell responses were detectable around this time [43,49]. Enhanced Tfh differentiation was associated in both models with a substantial increase by 6 days *p*.*i*., in ICOS expression by splenic CD4^+^ T-cells (Fig 5A & 5B). ICOS expression by CD4^+^ T cells facilitates interaction with ICOSL-expressing B-cells at the periphery of B-cell zones, and sustains Tfh cells within B-cell follicles [19,22,23]. Therefore, we examined the impact of IFNAR1-signalling on CD4^+^ T-cell localization within the spleen (Fig 5C). At day 5 *p*.*i*. we observed higher densities of ICOS^+^ T-cells at the T/B border in *Ifnar1* ^*-/-*^ mice compared to WT controls (Fig 5C), with a similar although more modest effect within B-cell follicles themselves (Fig 5C). Therefore, our data suggested that IFNAR1-signalling limited Tfh cell differentiation and the localization of activated CD4^+^ T-cells adjacent to and within B-cell follicles.

**Fig 6:**
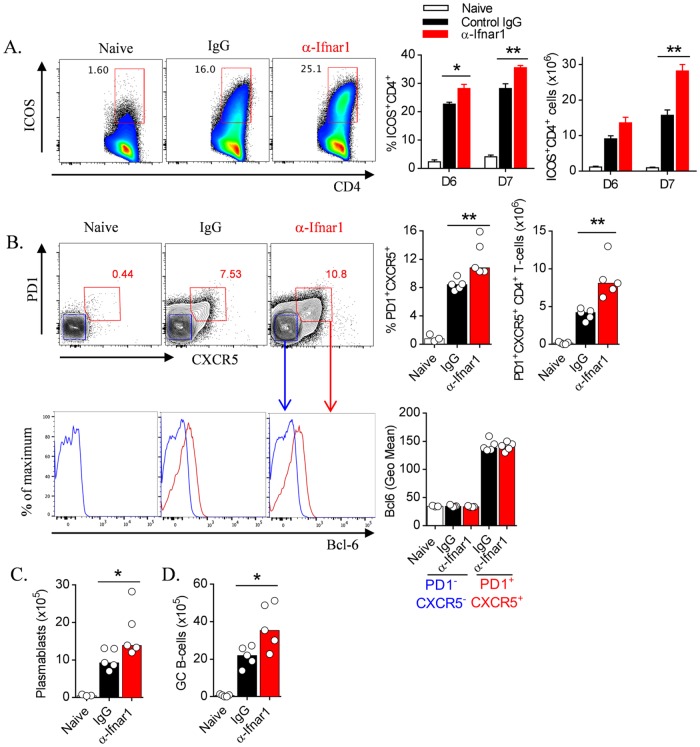
Antibody-mediated IFNAR1 blockade boosts humoral immune responses during blood-stage infection. WT mice (n = 5/group) were treated with anti-IFNAR1 blocking antibody (α-Ifnar1) or control IgG prior to and during infection with *Pc*AS. (A) Representative FACS plots (gated on CD4^+^ TCRβ^+^ live singlets), proportions and absolute numbers of splenic ICOS^+^ CD4^+^ T cells in naïve and infected mice on days 6 and 7 *p*.*i*. (B) Representative FACS plots (gated on CD4^+^ TCRβ^+^ live singlets), proportions and numbers of splenic Tfh cells (as PD1^+^CXCR5^+^ CD4^+^ T cells) in naïve and infected and antibody-treated mice, 7 days *p*.*i*. Bcl-6 expression is also shown in histograms for Tfh (PD1^+^CXCR5^+^; red gate) and non-Tfh cells (PD1^-^CXCR5^-^; blue gate), alongside Geometric Mean Bcl-6 expression by these populations in individual mice. (C and D) Numbers of splenic (C) plasmablasts and (D) GC B cells in naïve, and infected and treated mice, 7 days *p*.*i*. Data representative of 2 independent experiments. Mann-Whitney U test *P<0.05; **P<0.01.

To rule out possible developmental or immune homeostatic defects in *Ifnar1* ^*-/-*^ mice accounting for phenomena described above, we next treated *Pc*AS-infected WT mice with an IFNAR1-blocking antibody [42,43]. IFNAR1-blockade enhanced ICOS expression on CD4^+^ T-cells (Fig 6A), boosted Tfh cell responses but not Bcl-6 expression (Fig 6B), and increased early plasmablast and GC B-cell formation (Fig 6C & 6D). Together, these data supported the hypothesis that IFNAR1-signalling regulated ICOS expression by CD4^+^ T-cells, limited Tfh differentiation, and restricted splenic B-cell responses.

### IFNAR1-signalling limits both Th1 and Tfh differentiation

**Fig 7:**
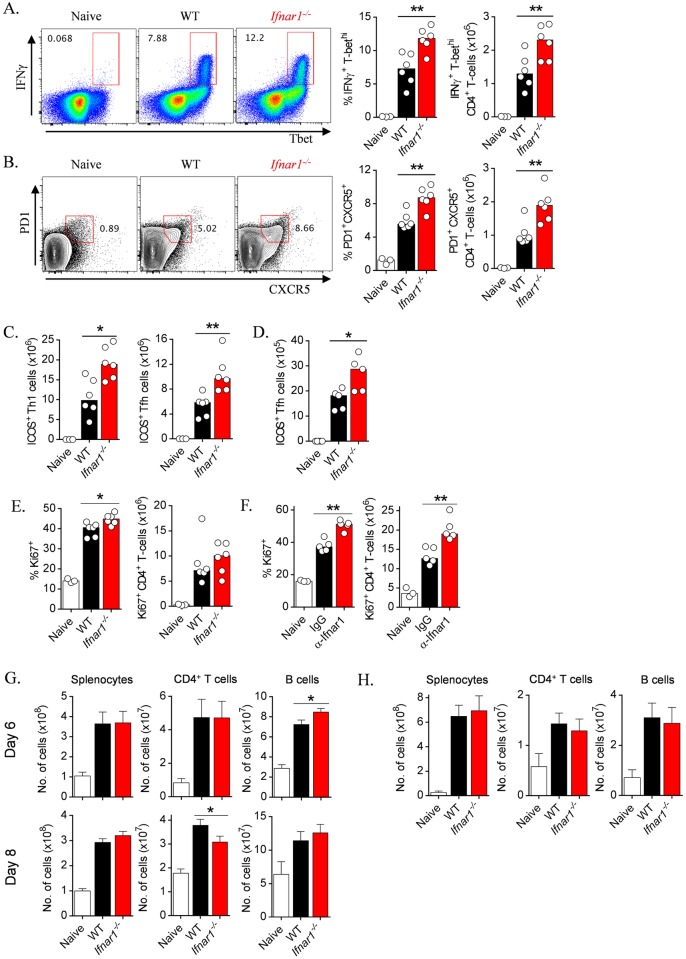
IFNAR1-signalling simultaneously limits splenic Th1 and Tfh cell responses. (A & B) Representative FACS plots (gated on CD4^+^ TCRβ^+^ live singlets), proportions and absolute numbers of splenic (A) Th1 (IFNγ^+^ Tbet^+^) and (B) emerging Tfh (PD1^+^ CXCR5^+^) cells in WT *and Ifnar1* ^*-/-*^ mice (n = 6/group), 6 days *p*.*i* with *Pc*AS. Data representative of two independent experiments. (C) Numbers of splenic ICOS^+^ Th1 cells (Tbet^+^ IFNγ^+^ CD4^+^ T cells) and Tfh cells (PD1^+^CXCR5^+^ CD4^+^ T cells) 6 days *p*.*i*. with *Pc*AS. (D) Numbers of ICOS^+^ Tfh cells (PD1^+^CXCR5^+^ CD4^+^ T cells), 16 days *p*.*i*. with *Py*17XNL infection. (E) Proportions and absolute numbers of splenic CD4^+^ T-cells expressing Ki-67 in naïve, WT and *Ifnar1* ^*-/-*^ mice 6 days *p*.*i*. with *Pc*AS. (F) Proportions and absolute numbers of splenic CD4^+^ T-cells expressing Ki-67 in naïve mice, and WT mice, 6 days *p*.*i*. with *Py*17XNL and treatment with α-IFNAR1 or Control IgG. (G) Absolute numbers of splenocytes, CD4^+^ T-cells and B-cells, in WT naïve, infected WT and *Ifnar1* ^*-/-*^ mice 6 days (n = 17–18, pooled from three independent experiments (n = 5–6 per expt)) and 8 days (n = 29, pooled from five experiments (n = 5–6 per expt)) *p*.*i*. with *Pc*AS. (H) Absolute numbers of splenocytes, CD4^+^ T-cells and B-cells in WT naïve, infected WT and *Ifnar1* ^*-/-*^ mice, 16 days *p*.*i*. with *Py*17XNL (n = 17–18, pooled from three experiments (n = 5–6 per expt)) Mann-Whitney U-test **P<0.01, *P<0.05.

Elevated IFNγ responses during blood-stage malaria have been associated with impaired humoral immune responses [17,50,51]. Since IFNAR1-signalling had suppressed IFNγ production by Th1 cells in our previous reports using *Pc*AS and *Pb*ANKA infection [42,43], we next determined the concurrent effect of IFNAR1-signalling on Th1 and Tfh differentiation. On day 6 *p*.*i*. with *Pc*AS, as expected, *Ifnar1* ^*-/-*^ mice exhibited increased Th1 differentiation compared to WT controls [43] (Fig 7A). Importantly, emerging Tfh responses at day 6 *p*.*i*. were also higher in *Ifnar1* ^*-/-*^ mice compared to WT controls (Fig 7B). This indicated that IFNAR1-signalling had simultaneously limited both Th1 and Tfh cell formation, rather than skewed CD4^+^ T cell responses to either helper subset. Moreover, IFNAR1-mediated regulation of ICOS expression in CD4^+^ T cells was observed in both the Th1 and emerging Tfh compartments during *Pc*AS infection (Fig 7C); and IFNAR1-signalling also reduced ICOS^+^ Tfh cell numbers during *Py*17XNL infection (Fig 7D).

Next, given the simultaneous effect of IFNAR1-signalling on Th1 and Tfh differentiation, we examined whether IFNAR1-signalling exerted a generalized effect on CD4^+^ T-cells. At day 6 *p*.*i*. during *Pc*AS infection, the proportion of CD4^+^ T-cells that were proliferating, as assessed by Ki-67-staining (Fig 7E), was modestly higher in *Ifnar1* ^*-/-*^ mice compared to WT controls. Similarly, in α-IFNAR1-treated *Py*17XNL-infected mice at day 6 *p*.*i*., CD4^+^ T-cells were more proliferative compared to isotype-treated controls (Fig 7F). We also examined the effect of IFNAR1-signalling on CD4^+^ T-cell activation using the markers CD11a and CD49d [4,41]. At day 8 *p*.*i*. with *Pc*AS, the proportion of CD4^+^ T-cells co-expressing CD11a and CD49d was modestly higher in *Ifnar1* ^*-/-*^ mice compared to WT controls, although absolute numbers in the spleen were not different (S3A Fig). We also studied *Py*17XNL-infected mice at day 6 *p*.*i*. after treatment with α-Ifnar1. We noted again that CD4^+^ T-cells were more activated in α-Ifnar1-treated mice compared to isotype-treated controls (S3B Fig). Taken together, our data suggested that IFNAR1-signalling limited early CD4^+^ T-cell activation and proliferation.

Finally, we observed no substantial differences in the cellularity of the spleen, or bulk CD4^+^ T cell or B-cell numbers, in WT versus *Ifnar1* ^*-/-*^ mice during *Pc*AS infection (Fig 7G), *Py*17XNL infection (Fig 7H), or in un-infected mice (S3C Fig). These data support the idea that any increases in Th1 or Tfh cells observed in our studies were not due to generalized increases in spleen cellularity, but instead were associated with specific regulation of CD4^+^ T cell activation and proliferation. Taken together, our data revealed that IFNAR1-signalling simultaneously regulated Th1 and Tfh cell formation, which was associated with restricted CD4^+^ T-cell activation, proliferation and ICOS expression.

### IFNAR1-signalling via cDCs limits Tfh cell and GC B-cell responses

**Fig 8:**
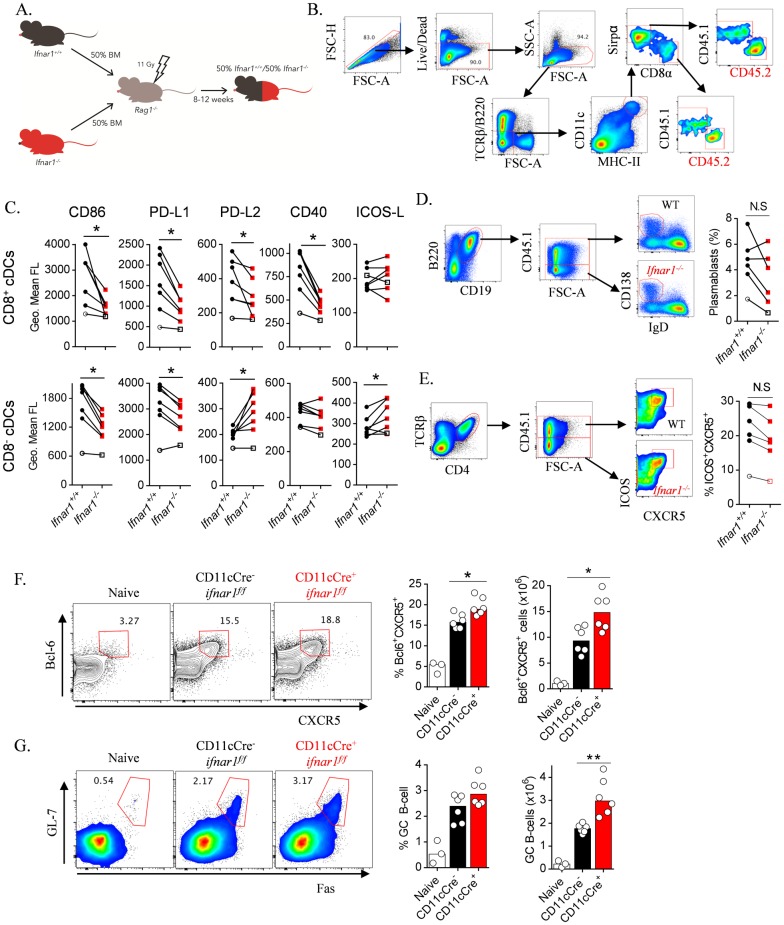
IFNAR1-signalling via cDCs limits Tfh and GC B-cell responses. (A) Schematic for the mixed bone marrow chimeric approach: 50:50 mixed WT (*Ifnar1* ^*+/+*^; CD45.1) and *Ifnar1* ^*-/-*^ (CD45.2) bone marrow was transferred into lethally-irradiated *rag1* ^*-/-*^ mice (to avoid potential issues with residual radio-resistant T- and B-cells), and subsequently left for 8–12 weeks prior to infection studies. (B) Gating strategy for splenic WT (CD45.1^+^) and *Ifnar1* ^*-/-*^ (CD45.2^+^) CD8^+^ (TCRβ^-^ B220^-^ CD11c^hi^ MHC-II^hi^ CD8α^+^) and CD8^-^ (TCRβ^-^ B220^-^ CD11c^hi^ MHC-II^hi^ Sirpα^+^ CD8^-^) conventional DCs. (C) Paired analysis between splenic WT and *Ifnar1* ^*-/-*^ cDC subsets in individual mice for cell-surface expression of CD86, PD-L1, PD-L2, CD40 and ICOS-L, 2 days *p*.*i*. with *Py*17XNL. Data representative of two independent experiments. Statistics: Wilcoxon test, *P<0.05. (D) Representative FACS plots and proportions for WT (CD45.1^+^) *Ifnar1* ^*+/+*^ and *Ifnar1* ^*-/-*^ plasmablasts, 7 days *p*.*i*. with *Pc*AS infection. Data representative of 2 independent experiments. Statistics: Wilcoxon test. (E) Representative FACS plots and proportions for WT (CD45.1^+^) *Ifnar1* ^*+/+*^ and *Ifnar1* ^*-/-*^ CD4^+^ T cells expressing ICOS and CXCR5, 7 days *p*.*i*. with *Pc*AS infection. Experiment performed once. Statistics: Wilcoxon test, *P<0.05. (F) Representative FACS plots (gated on CD4^+^ TCRβ^+^ live singlets), proportions and absolute numbers of splenic Tfh cells and (G) splenic GC B-cells (gated on B220^+^ CD19^+^ live singlets) in CD11cCre^+/-^ *ifnar1* ^*f/f*^ and CD11cCre^-/-^ *ifnar1* ^*f/f*^ littermates (n = 6) (WT naïve mice as staining controls), on day 6 *p*.*i*. with *Py*17XNL; Statistics: Mann-Whitney U-test **P<0.01, *P<0.05. Data representative of 2 independent experiments.

We next determined cell types in which IFNAR1-signalling had occurred, and focused on cDCs given our previous work using lethal *Pb*ANKA infection [42]. We generated mixed bone marrow chimeric mice as before [42], which harboured equal proportions of WT and *Ifnar1* ^*-/-*^ splenic cDCs (Fig 8A). These mice were infected with *Py*17XNL, and expression of co-stimulatory molecules was assessed at the peak of the cDC response (at 2 days *p*.*i*.) on splenic WT and *Ifnar1* ^*-/-*^ cDC subsets (Fig 8B). Up-regulation of CD86 in particular, which we previously found was dependent on IFNAR1-signalling in cDC [42], was again substantially impaired in *Ifnar1* ^*-/-*^ CD8^+^ and CD8^-^ cDC subsets compared to WT cDCs (Fig 8C). A similar pattern of expression was seen for PD-L1, while we observed minimal changes in ICOSL expression (Fig 8C). Interestingly, we also observed cDC subset-specific effects, with PD-L2 restrained by IFNAR1-signalling in CD8^-^ cDC subsets, and CD40 expression mediated by IFNAR1-signalling in CD8^+^ cDCs (Fig 8C). Similar observations were also made for CD86, CD40 and ICOSL during *Pc*AS infection (S4 Fig). Taken together, our data demonstrated that similar to lethal *Pb*ANKA infection [42], IFNAR1-signalling in splenic cDC subsets influenced their upregulation of co-stimulatory molecules during non-lethal blood-stage *Plasmodium* infection.

To study the possibility of IFNAR1-signalling in B-cells, we next examined early plasmablast responses in WT:*Ifnar1* ^*-/-*^ mixed bone marrow chimeric mice during *Pc*AS infection. At day 7 *p*.*i*., there was no consistent increase in plasmablast formation by *Ifnar1* ^*-/-*^ cells compared to WT counterparts (Fig 8D), suggesting that IFNAR1-signalling in B-cells played no major role in regulating plasmablast differentiation. Moreover, in these same mice equal proportions of WT and *Ifnar1* ^*-/-*^ CD4^+^ T cells upregulated ICOS and CXCR5 (Fig 8E). This suggested that restriction of ICOS expression in emerging Tfh cells was not mediated by IFNAR1-signalling directly to CD4^+^ T cells.

Finally, we explored *in vivo* the effect of IFNAR1-signalling in cDC on emerging humoral responses in the spleen. CD11cCre^+/-^ *ifnar1* ^*f/f*^ mice and CD11cCre^-/-^ *ifnar1* ^*f/f*^ littermate controls were infected with *Py*17XNL, and splenic Tfh and GC B-cell differentiation assessed 6 days *p*.*i*. (Fig 8F and 8G). We noted that IFNAR1-signalling in CD11c^hi^ cells limited both Tfh (Fig 8F) and early GC B-cell (Fig 8G) differentiation. Taken together, our data strongly suggested that regulation of humoral immune responses by IFNAR1-signalling was mediated through cDCs, not B-cells or CD4^+^ T cells.

### IFNAR1-deficiency boosts humoral immunity in an ICOS-dependent manner

**Fig 9:**
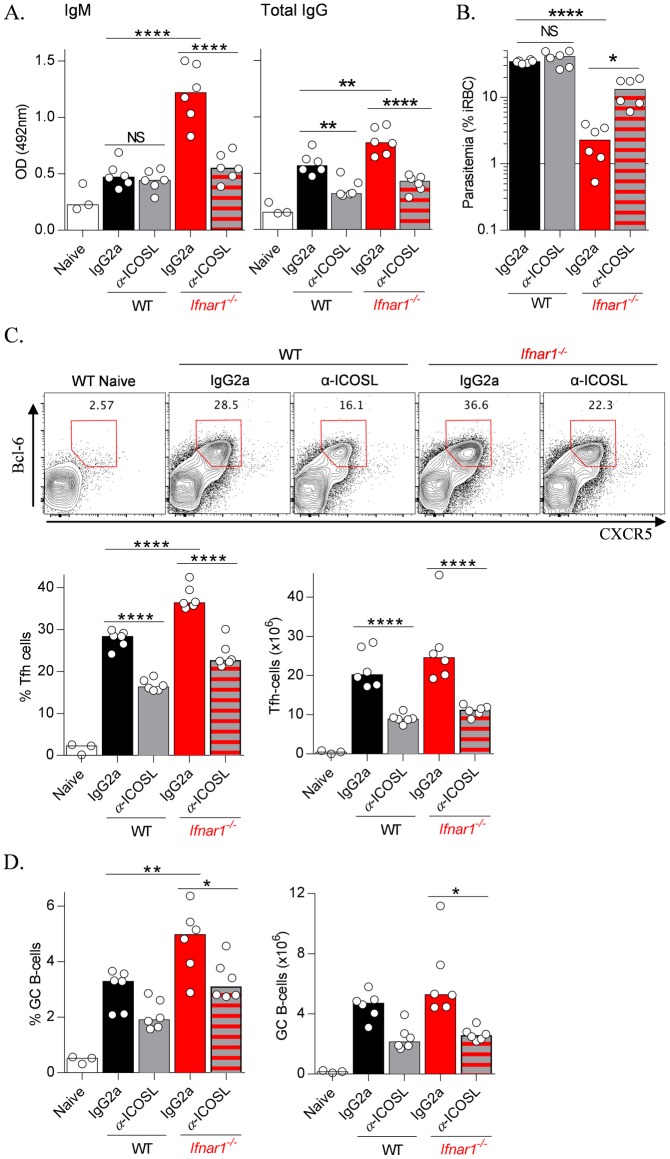
IFNAR1-deficiency boosts humoral immunity via enhanced ICOS-signalling. WT and *Ifnar1* ^*-/-*^ mice (n = 6/group), infected with *Pc*AS, and treated with αICOSL (100μg) or control IgG2a throughout infection (days 0, 2, 4 and 6 *p*.*i*.), were assessed on day 8 *p*.*i*. for (A) parasite-specific IgM and IgG, as well as (B) Parasitemia. (C&D) WT and *Ifnar1* ^*-/-*^ mice were infected with *Pc*AS, and treated as in (A & B) with α-ICOSL or control IgG2a: (C) Representative FACS plots (gated on CD4^+^ TCRβ^+^ live singlets), proportions and absolute numbers of splenic Tfh cells, and (D) proportions and absolute numbers of GC B-cells (gated on B220^+^ CD19^+^ live singlets) on day 6 *p*.*i*. Statistics: One-way ANOVA, Tukey’s test for multiple comparisons, *P<0.05; **P<0.01; ****P<0.0001. (A) & (B) representative of two independent experiments. (C) & (D) representative of three independent experiments.

Finally, we sought to explore molecular mechanisms by which IFNAR1-deficiency accelerated the onset of humoral immunity, and hypothesized a role for ICOS-signalling. To test this, we adopted a recent approach in which α-ICOSL blocking antibody was used to reduce ICOS-signalling in mice exhibiting higher than WT levels of ICOS [28]. We treated *Pc*AS-infected *Ifnar1* ^*-/-*^ and WT mice with a moderate dose of α-ICOSL blocking antibody (100μg) and at day 8 *p*.*i*. examined antibody production and parasitemia. We saw no effect on early parasite-specific IgM production or parasitemias in WT mice (Fig 9A & 9B), with some impairment of emerging parasite-specific IgG responses (Fig 9A & 9B). In contrast, ICOS-signalling blockade in *Ifnar1* ^*-/-*^ mice abrogated any improvements in parasite-specific IgM or IgG production (Fig 9A), and importantly, impaired parasite control compared to control-treated *Ifnar1* ^*-/-*^ mice (Fig 9B). Consistent with these observations, we also noted that enhanced Tfh cell and GC B-cell formation in *Ifnar1* ^*-/-*^ mice was strongly dependent on ICOS-signalling (Fig 9C & 9D). Finally, ICOS blockade in *Ifnar1* ^*-/-*^ mice significantly reduced serum IFNγ levels compared to control-treated *Ifnar1* ^*-/-*^ mice (S5 Fig), consistent with enhanced ICOS-signalling during IFNAR1-deficiency being supportive of Th1 differentiation. Taken together, our data support a model in which IFNAR1-signalling limited humoral immune responses and parasite control by regulating ICOS-signalling.

## Discussion

Here, using two mouse models of non-lethal blood-stage malaria, we have provided evidence that the onset of protective humoral immunity to *Plasmodium* can be influenced by an innate cytokine signalling pathway, in this case Type I Interferon-signalling via IFNAR1. Therefore, we have demonstrated for the first time that innate immune cytokines can limit the onset of antibody production and B-cell responses to *Plasmodium*. Moreover, we found that release from IFNAR1-mediated immune-regulation enhanced humoral immune responses and parasite control in a manner dependent upon ICOS-signalling.

Although parasite-specific antibodies can control blood-stage *Plasmodium* parasite numbers *in vivo*, protective humoral immunity can take months or years to develop in humans. Reasons for this have focused on parasite mechanisms such as antigenic variation, rather than possible sub-optimal host immune responses. IFN-I responses have been well documented in malaria patients [38,52,53]. Polymorphisms in the *Ifnar1* gene have been associated with reduced risk of severe malaria [38,52]. Although the location of polymorphisms did not indicate the direction of their effect on IFNAR1-signalling, the implication was that changes in IFNAR1-signalling could mediate improved parasite control. Indeed, our recent work using C57BL/6J mice suggested that IFNAR1-signalling via IRF7 but not IRF3 limited parasite control during *Pc*AS infection [42,43], although other recent work, using a different route of infection [54] or a different genetic background [55] suggested more modest roles for IFN-I-signalling in mice. These different outcomes suggest possible context-dependent effects for IFN-I-signalling during *Pc*AS infection.

It is likely that mouse models of malaria will be informative for studies of cytokine-mediated effects on humoral immunity, as recently epitomized in studies of T cell-derived IL-21 [6]. These reports and our new data complement studies of T-cell co-stimulatory molecules, such as PD-1 and LAG-3, and strengthen the idea that multiple molecular targets could be harnessed to boost humoral immunity to malaria [4,17]. Whether such strategies are more applicable to natural exposure events or vaccination scenarios remains to be studied. In addition, while we found no overt evidence of increased immune-pathology in *Ifnar1* ^*-/-*^ or α-Ifnar1 treated mice, it will be important to better test whether manipulation of IFNAR1-signalling triggers unwanted adverse events, as previously reported for IL-10- or IL-27-deficiency [56,57].

Much of our current understanding of cytokine-mediated control of humoral immunity derives from studies of viral infection or experimental immunization in mice [33,58,59,60,61]. However, it remains difficult to infer from these studies how cytokine-signalling impacts upon antibody responses during parasitic infection. In a recent report, Perez-Mazliah *et al*., demonstrated a pivotal role for T cell-derived IL-21 in mediating GC B-cell responses and IgG class switching but not for generating Tfh cells during blood-stage *Plasmodium* infection [6]. Our demonstration that IFNAR1-signalling restricts the onset of humoral immunity to malaria is the first description of cytokine-mediated suppression of Tfh, GC B-cell and plasmablast formation during parasitic infection. More generally, while the majority of viral and experimental immunization studies highlight a positive role for IFNAR1-signalling in driving humoral immunity, our data emphasizes that depending on the experimental context, IFNAR1-signalling can also limit humoral immune responses. Our previous studies using a lethal model of malaria demonstrated that IFNAR1- signalling occurred via cDC, which resulted in potent suppression of Th1-immunity, and was associated with effects on PDL1, PDL2 and IL-10 expression by cDC subsets [42]. However, the lethal model was not suitable for studying humoral immunity, since mice became moribund within the first week of infection. Here, using non-lethal models, we similarly revealed that IFNAR1-signalling proceeding via cDCs, but not B- or T-cells, regulated the generation of Tfh and GC B-cell responses. Given that cDCs are critical for early Tfh differentiation and initiation of humoral immune responses [18,62,63], we propose that IFNAR1-signalling within splenic cDC regulates CD4^+^ T-cell proliferation, ICOS expression, and Th1 and Tfh differentiation.

One question that remains to be answered is how the interaction between cDC and CD4^+^ T cells is altered at a molecular level by IFNAR1-signalling. This work and our previous study using *Pb*ANKA infection [42] both revealed multiple changes in the co-stimulatory landscape on the surface of cDC, including a substantial shift in the ratio of PDL1:PDL2 expression on CD8^-^ cDC. Given a recent report that PDL2-signalling can compete against the regulatory effects of PDL1, and indeed, can protect against experimental malaria [64], we speculate that IFNAR1-signalling in cDC regulates CD4^+^ T cell activation by favouring PDL1-signalling over protective PDL2 signals.

It was recently reported during LCMV infection that IFN-I promoted Th1 responses, which then suppressed Tfh and GC B-cell responses via IFNγ [44]. A more recent report using a mouse model of malaria demonstrated that therapeutic release from PD-1 exhaustion, coupled with stimulation via OX40 dramatically increased IFNγ production by Th1 cells, which destabilized Bcl-6 in established Tfh cells and resulted in defective humoral immune responses [17]. Elsewhere, circulating ‘Th1-like’ Tfh cells were associated with impaired humoral responses in children living in malaria endemic areas [50]. Most recently, Ryg-Cornejo *et al*., implicated a combined effect of IFNγ and TNF in driving sub-optimal humoral responses during severe malaria infections in mice [51]. Together, these data suggest that Th1 responses might interfere with Tfh responses. However, in our studies, we found that deficiency in IFNAR1-signalling triggered a concurrent increase in both Th1 and Tfh responses. This apparent discrepancy between our work and recent studies could be explained by the differential kinetics of the elevated Th1 responses in our respective studies. For instance, OX40- and PD1-targeted therapy was initiated from the second week of infection, a period of time after which Th1/Tfh priming would likely have occurred [17]. In our studies, elevated Th1 responses, as a result of IFNAR1-deficiency, occurred relatively transiently within the first week of infection. Therefore, we speculate that elevated Th1 responses in IFNAR1-deficient mice did not destabilize Tfh responses because they occurred early and were not prolonged.

Data from this and our previous studies [42,43,49] support a model in which abrogation of IFNAR1-signalling has the dual effect of boosting Th1 responses and antibody-production. These effects associated with improved control of *Py*17XNL, *Pc*AS and *Pb*ANKA parasites; yet crucially we have not yet demonstrated a causal link between IFNAR1-deficiency and improved antibody-mediated parasite control. Nevertheless, we propose that depending upon host genetic background and that of the infecting *Plasmodium* species, IFNAR1-signalling can obstruct parasite control via a number of mechanisms including the regulation of Th1 cells and/or antibody production.

Numerous studies across a range of experimental systems have established a pivotal role for ICOS signalling in CD4^+^ T-cells in mediating T-cell dependent humoral immunity, via effects on Tfh generation, maintenance and trafficking [18,19,23,65]. The importance of ICOS-signalling is further highlighted by the existence of multiple layers of regulation within the T-cell for limiting its expression, for example via Roquin 1, Roquin 2 and microRNA146a [26,27,28]. To date, however, evidence of T-cell extrinsic mechanisms for controlling ICOS levels on CD4^+^ T-cells has been limited. Our data reveals the existence of a cytokine signalling pathway, mediated by IFNAR1 which serves to limit the level of ICOS on CD4^+^ T-cells. Currently, the mechanism by which ICOS levels are modulated in our models by IFNAR1-signalling is unclear, but could theoretically involve Roquin1, Roquin 2 or microRNA146a.

Although we observed substantial early ICOS expression by activated CD4^+^ T-cells in our models, we noted minimal change in ICOSL levels on cDC (Fig 8C). This might suggest that boosting ICOS expression by CD4^+^ T-cells did not encourage further interaction with splenic cDC. Instead, given that IFNAR1-deficiency increased the frequency of ICOS^+^ T-cells close to and within B-cell follicles, which were themselves essential for supporting Tfh responses (S2 Fig), and since ICOSL-expressing B-cells are located at the periphery of B-cell follicles [22], we propose that IFNAR1-signalling limits ICOS-mediated positioning of emerging Tfh cells adjacent to and within B-cell follicles during *Plasmodium* infection. However, further experiments will be required to examine the effect of ICOS-signalling on CD4^+^ T-cell trafficking in the spleen during experimental malaria.

In this study, we discovered that IFNAR1-deficiency accelerated early production of parasite-specific IgM and IgG. If such mechanisms could be induced in humans, this might improve control of parasite numbers and prevention of clinical malaria. However, the duration of this effect would require scrutiny, since in our mouse models, antibody levels normalized between *Ifnar1* ^*-/-*^ and WT mice by the seventh week of infection. Furthermore, whether acceleration in early antibody production would increase the rate of acquisition of immunity to clinical malaria is also unclear, particularly given the phenomenon of antigenic variation. However, we speculate that if subsequent infections were sufficiently similar from an antigenic perspective to previously encountered parasites, short-term elevations in parasite-specific antibodies could be beneficial. Finally, although we focused on studying the magnitude of parasite-specific antibody responses within a given antibody sub-class, neither antibody affinity nor avidity was examined. Therefore, further experimentation will be needed to determine whether beneficial changes in antibody affinity and avidity can be brought about via cytokine modulation.

In summary, we have demonstrated here that early cytokine-signalling during infection influences parasite-specific antibody production, and associated GC B-cell, plasmablast and Tfh differentiation in two models of non-lethal blood-stage malaria. Our work suggests that antibody-mediated immunity to malaria might be improved by targeting cytokine-signalling pathways, particularly in the context of natural infection.

## Experimental Procedures

### Ethics statement

All animal procedures were approved by the QIMR Berghofer Medical Research Institute Animal Ethics Committee, under approval numbers A02-633M and A1503-601M, in accordance with the “Australian Code of Practice for the Care and Use of Animals for Scientific Purposes” (Australian National Health and Medical Research Council).

### Mice

Female C57BL/6J and congenic CD45.1^+^ C57BL/6J mice (6–12 weeks old) were purchased from Australian Resource Centre (Canning Vale, Western Australia) and maintained under conventional conditions. C57BL/6J *Ifnar1* ^*-/*-^ mice were maintained in-house at QIMR Berghofer Medical Research Institute. Mixed bone marrow (BM) chimeric mice were prepared as previously described [42]. Briefly, 2x10^6^ fresh syngeneic BM cells from femurs of CD45.1^+^ wild type and CD45.2^+^ *Ifnar1* ^*-/*-^ mice, mixed at a 50:50 ratio were intravenously (i.v.) transferred into lethally irradiated [11Gy (137Cs source)] C57BL/6J *Rag1* ^-/-^ recipient mice. These mice were then treated for 14 days with Baytril (Provet) in drinking water. Engraftment was assessed after 8–12 weeks by flow cytometry. BM Chimeric mice were infected 12 weeks after bone marrow transplantation.

### Parasites, infections and parasitemia

Non-lethal *Plasmodium yoelii* 17XNL (*Py17XNL*) and *Plasmodium chaubadi chaubadi* AS (*Pc*AS) parasites were used following one *in vivo* passage in wild type C57BL/6J mice. Using parasitized red blood cells (pRBC) that were obtained from frozen/thawed stabilates, mice were infected i.v. with either 10^4^ pRBCs (*Py*17XNL) or 10^5^ pRBCs (*Pc*AS). Blood parasitemia was measured in Diff-Quick (Lab Aids, Narrabeen, NSW, Australia) or giemsa-stained thin blood smears obtained from tail bleeds. Alternatively, a modified protocol of a previously established flow cytometric method was employed to measure parasitemia more rapidly [66]. Briefly, a single drop of blood, from a tail bleed or cardiac puncture, was diluted and mixed in 250μl RPMI containing 5U/ml heparin sulphate. Diluted blood was simultaneously stained with Syto84 (5μM; Life Technologies) to detect RNA/DNA, and Hoechst33342 (10μg/ml; Sigma) to detect DNA, for 30 minutes, in the dark at room temperature. Staining was quenched with 10x volume of ice cold RPMI, and samples were immediately analysed by flow cytometry, using a BD FACSCantoII analyser (BD Biosciences) and FlowJo software (Treestar, CA, USA). pRBC were readily detected as being Hoechst33342^+^ Syto84^+^, with white blood cells excluded on the basis of size, granularity and much higher Hoechst33342/Syto84 staining compared to pRBC.

### Preparation of crude parasite antigen

Crude antigen extract from *Py*17XNL or *Pc*AS-infected RBC was prepared using an adapted version of a previously described protocol [67,68]. Briefly, mice were infected with *Py*17XNL or *Pc*AS as described above. When parasitemias reached 20–30%, blood was collected by cardiac puncture into heparinized tubes. RBCs were washed once in RPMI at 1200rpm for seven minutes at room temperature, and then lysed using ultrapure water followed by four washes in ice-cold PBS at 16,000xg for 25 minutes at 4°C, as well as three cycles of freezing (two hours at -80°C) and thawing (30 minutes at room temperature). Extracts were also processed from RBCs of uninfected C57BL/6J mice, for use as negative controls in ELISA. The concentration of proteins in the purified extracts was determined by Bradford assay (Thermo Scientific). All extracts were stored at -80°C until use.

### Detection of parasite-specific serum antibodies by ELISA

Costar EIA/RIA 96-well flat bottom plates were coated overnight at 4°C with 2.5μg of soluble antigen/ml in bicarbonate coating buffer (pH9.6). Wells were washed three times (all washes in 0.005% Tween in PBS) and then blocked for 1hr at 37°C with 1% BSA in PBS. Wells were washed three times, 100ul of sera diluted 1/400, 1/800, 1/1600 or 1/3200 was added and incubated for 1hr at 37°C. Following six washes, wells were incubated in the dark with biotinylated anti-IgM, total IgG, IgG1, IgG2b and IgG3 (Jackson ImmunoResearch) for 1hr at room temperature. Unbound antibodies were washed off (six times) prior to incubating wells in the dark with streptavidin HRP (BD pharmagen) for 30 minutes at room temperature. Wells were washed six times prior to development (100μl, OPD; Sigma-Aldrich) for five minutes in the dark before termination with an equal volume of 1M HCl. Absorbance was determined at 492nm using a Biotek synergy H4 ELISA plate reader (Biotek, USA). Data were analysed using Gen5 software (version 2) and GraphPad Prism (version 6).

### Flow Cytometry and Antibodies

Spleen mononuclear cells were prepared as previously described [69]. For studies of cDCs, spleens were treated with deoxyribonuclease I (0.5mg/ml; Worthington Biochemical) and collagenase type 4 (1mg/ml; Worthington Biochemical) for 25 minutes at room temperature, in order to ensure maximal recovery of splenic cDCs. Fluorescently conjugated monoclonal antibodies, anti-mouse B220-Alexa Fluor 700 (RA3-6B2), B220-Pacific blue (RA3-6B2), CD19-FiTC (6D5), CD138-BV605 (281–2), IgD-APCCy7 (11-26c.2a), IgM-PECy7 (RMM-1), TCRβ-Alexa Fluor 700 (H57-597), TCRβ-APC/Cy7 (H57-597), CD4-BV605 (RM4-5), IFNγ-BV421 (XMG1.2), ICOS-PE (7E.17G9), Streptavidin-PE/Cy7, CD45.1-FiTC (A20), CD45.2-Alexa Fluor 700 (104), CD11c-APC (N418), MHCII-Pacific blue (M5/114.15.2), CD8α-PE/Cy7 (53–6.7), CD40-PE (1C10), CD80-PE (16-10A1), CD86-PE (GL1), ICOSL-PE (B7-RP1), PDL1-PE (MIH5), PDL2-PE (TY25), CD49d-Biotin (R1-2), CD11a-FiTC (M17/4), Ki-67-PE (16A8) and Zombie Aqua fixable viability dye were purchased from Biolegend (San Diego, CA). Anti-mouse CD95/Fas-BV421 (Jo2), CXCR5-biotin (2G8), and Bcl6-PerCP/Cy5.5 (K112-91) were purchased from BD Biosciences (Franklin Lakes, NJ). Anti-mouse T-bet-APC (eBio4B10), GL-7-APC (GL-7) and PD1-APC/Cy7 (J43) were purchased from eBioscience. Cell surface and intracellular IFNγ, T-bet and Bcl6 staining was performed as previously described [69,70].

### *In vivo* cell depletion and blockade using monoclonal antibodies

Anti-CD4 depleting monoclonal antibody (clone GK5.1) and its isotype control were administered in 0.1mg doses, via intravenous (*i*.*v*.) injection in 200μl 0.9% NaCl (Baxter) one day before infection. CD4^+^ T-cell depletion was confirmed in PBMC over the first four days of infection, and absolute numbers subsequently remained >99% depleted in the spleen at day 10 *p*.*i*. compared to isotype-treated controls. For ICOSL blockade in Fig 2A–2F, α-ICOSL (clone HK5.3, BioXCell) and its isotype control (IgG2a, clone 2A3, BioXCell) were administered in 0.2mg doses, via *i*.*v*. injection in 200μl 0.9% NaCl (Baxter) one day prior to infection, and then every three days for up to 15 days *p*.*i*. (and for up to 21 days in Fig 2G). For ICOSL blockade experiments in Fig 9 and S5 Fig, α-ICOSL (clone MIL5733) and its isotype control (IgG2a, clone 1D10) were generated in-house and administered in 0.1mg doses, via *i*.*v*. injection in 200μl 0.9% NaCl (Baxter) one day prior to infection, and then every two days for up to 6 days *p*.*i*. (In Fig 9, the 100μg dose was employed since preliminary experiments indicated that 200μg reduced Tfh cell responses by ~65%, 100μg reduced Tfh responses by 50%, and 25μg had no effect by day 8 *p*.*i*. with *Pc*AS). For B-cell depletion, anti-CD20 (clone 5D2) or its isotype control antibody were produced and kindly provided by Genentech and administered in a single 0.5mg dose via *i*.*p*. injection in 200μl 0.9% NaCl (Baxter), five days prior to infection. For Ifnar1 blockade, α-Ifnar1 blocking monoclonal antibody (clone MAR1-5A3; Leinco Technologies Inc) and its isotype control mAb were administered in 0.1mg doses, via *i*.*p*. injection in 200μl 0.9% NaCl (Baxter) on the day of infection, and subsequently on days 2, 4 and 6 *p*.*i*.

### Confocal microscopy analysis

Confocal microscopy was performed on 10–20 μm frozen spleen sections as previously described [71,72]. Briefly, tissues from infected and un-infected mouse spleens were snap frozen in embedding optimal cutting temperature (OCT) medium (Sakura) and stored at -80°C until use. Sections were fixed in ice-cold acetone for 10 minutes prior to labelling with antibodies against CD3-Biotin (clone-17A2), B220-PE (clone-RA3-6B2) and ICOS-APC (clone-C398.4A). Anti-CD3 was detected by streptavidin conjugated to Alexa Fluor 594. All antibodies were obtained from Biolegend (San Diego, CA). DAPI was used to aid visualization of white pulp areas. Samples were imaged on a Zeiss 780-NLO laser-scanning confocal microscope (Carl Zeiss Microimaging) and data analysed using Imaris image analysis software, version 8.1.2 (Bitplane). Cells were identified using the spots function in Imaris, with thresholds <10μm and intensities <150. T-B borders were defined by the region between CD3^+^ cells closest to the B cell follicle and B220^+^ cells furthest into the T-cell zone. All objects were manually inspected for accuracy before data were plotted and analyzed in GraphPad Prism (version 6)

### Statistical analysis

Comparisons between two groups were performed using non-parametric Mann-Whitney (unpaired datasets) or Wilcoxon (paired datasets) tests. Where depicted, one-way or two-way ANOVA and Tukey’s post-test were employed for multiple comparisons among three or more groups. p< 0.05 was considered significant (p<0.05 = *; p<0.01 = **; p<0.001 = ***; P<0.0001 = ****). Survival graphs were assessed using Log-rank (Mantel-Cox) tests. Graphs depict mean values ± SEM, except where individual mouse data points are depicted, in which case median values are shown. All statistical analyses were performed using GraphPad Prism v6 or v7 software.

## Supporting Information

S1 Fig IFNAR1-signalling suppresses parasite control, plasmablast and GC B-cell responses during *Pc*AS infection. WT and *Ifnar1* ^*-/-*^ mice (n = 5–9) were infected with *Pc*AS. (A) A time-course analysis of parasitemia in WT (n = 9/group) and *Ifnar1* ^*-/-*^(n = 6/group) mice Data representative of three independent experiments. Statistics: Mann-Whitney U test, **P<0.01, *P<0.05. (B&C) Representative FACS plots (gated on B220^+^ CD19^+^ live singlets), proportions and absolute numbers of (B) splenic plasmablasts (B220^+^CD19^+^IgD^lo^CD138^hi^) in naïve and infected mice, 6 days *p*.*i*., and (C) emerging splenic GC B-cells (B220^+^CD19^+^GL7^+^Fas^+^) in naïve and infected mice, 8 days *p*.*i*. Data in B&C representative of three independent experiments. Statistics: Mann-Whitney U test, **P<0.01.
(TIF)

S2 Fig B-cells are required for Tfh responses during *Pc*AS infection. WT mice (n = 5/group) were pre-treated with anti-CD20 depleting monoclonal antibody (α-CD20) or control-IgG before infection with *Pc*AS. Representative FACS plots (gated on CD4^+^ TCRβ^+^ live singlets), proportions and absolute numbers of splenic CD4^+^ T cells co-expressing PD1/CXCR5 and Bcl-6/CXCR5, 7 days *p*.*i*. Data representative of two independent experiments. Mann-Whitney U test, **P<0.01.
(TIF)

S3 Fig IFNAR1-signalling limits CD4^+^ T-cell activation during *Pc*AS and *Py*17XNL infection. (A). Representative FACS plots, proportions and numbers of splenic activated CD4^+^ T-cells (CD4^+^TCRβ^+^CD11a^+^CD49d^+^ live singlets) in WT and *Ifnar1* ^*-/-*^ mice (n = 6) 8 days *p*.*i*. with *Pc*AS. (B) Proportions and numbers of splenic activated CD4^+^ T-cells on day 6 *p*.*i*. with *Py*17XNL in WT mice (n = 5) treated with α-Ifnar1 or control IgG. (C) Total numbers of splenocytes, B-cells, GC B-cells and plasmablasts in un-infected WT *and Ifnar1* ^*-/-*^ mice (n = 5). Experiments performed once. Statistics: Mann-Whitney U test, *P<0.05; **P<0.01.
(TIF)

S4 Fig IFNAR1-signalling proceeds via CD11c^hi^ cDC during *Pc*AS infection. 50:50 WT (*Ifnar1* ^*+/*+^ CD45.1): *Ifnar1* ^*-/-*^ (CD45.2) mixed bone marrow chimeras (n = 6) were infected with *Pc*AS. Graphs show paired analysis between splenic WT and *Ifnar1* ^*-/-*^ cDC subsets in individual mice for cell-surface expression of CD86, CD40 and ICOS-L, on CD8α^+^ (TCRβ^-^ B220^-^ CD11c^hi^ MHC-II^hi^ CD8α^+^) and CD8α^-^ (TCRβ^-^B220^-^CD11c^hi^MHC-II^hi^Sirpα^+^CD8^-^) cDCs, 2 days *p*.*i*. (experiment performed once). White symbols denote un-infected control. Statistics: Wilcoxon test, *P<0.05.
(TIF)

S5 Fig ICOS-blockade limits serum IFNγ levels in *Pc*AS-infected *Ifnar1* ^*-/-*^ mice. *Ifnar1* ^*-/-*^ mice were infected with *Pc*AS, treated with α-ICOSL (100μg) or control IgG2a and assessed for IFNγ levels in serum on day 8 *p*.*i*. Data is pooled from two independent experiments showing similar results (n = 6/ experiment). Statistics: Mann-Whitney U test, **P<0.01.
(TIF)

## References

[1] Portugal S, Pierce SK, Crompton PD (2013) Young lives lost as B cells falter: what we are learning about antibody responses in malaria. J Immunol 190: 3039–3046. 10.4049/jimmunol.1203067 23526829 PMC3608210

[2] Langhorne J, Ndungu FM, Sponaas AM, Marsh K (2008) Immunity to malaria: more questions than answers. Nat Immunol 9: 725–732. 10.1038/ni.f.205 18563083

[3] Boyle MJ, Reiling L, Feng G, Langer C, Osier FH, et al (2015) Human antibodies fix complement to inhibit Plasmodium falciparum invasion of erythrocytes and are associated with protection against malaria. Immunity 42: 580–590. 10.1016/j.immuni.2015.02.012 25786180 PMC4372259

[4] Butler NS, Moebius J, Pewe LL, Traore B, Doumbo OK, et al (2012) Therapeutic blockade of PD-L1 and LAG-3 rapidly clears established blood-stage Plasmodium infection. Nat Immunol 13: 188–195. 10.1038/ni.2180 PMC3262959 22157630

[5] Cohen S, Mc GI, Carrington S (1961) Gamma-globulin and acquired immunity to human malaria. Nature 192: 733–737. 13880318 10.1038/192733a0

[6] Perez-Mazliah D, Ng DH, Freitas do Rosario AP, McLaughlin S, Mastelic-Gavillet B, et al (2015) Disruption of IL-21 signaling affects T cell-B cell interactions and abrogates protective humoral immunity to malaria. PLoS Pathog 11: e1004715 10.1371/journal.ppat.1004715 25763578 PMC4370355

[7] Raj DK, Nixon CP, Nixon CE, Dvorin JD, DiPetrillo CG, et al (2014) Antibodies to PfSEA-1 block parasite egress from RBCs and protect against malaria infection. Science 344: 871–877. 10.1126/science.1254417 24855263 PMC4184151

[8] Sabchareon A, Burnouf T, Ouattara D, Attanath P, Bouharoun-Tayoun H, et al (1991) Parasitologic and clinical human response to immunoglobulin administration in falciparum malaria. Am J Trop Med Hyg 45: 297–308. 1928564 10.4269/ajtmh.1991.45.297

[9] Seder RA, Chang LJ, Enama ME, Zephir KL, Sarwar UN, et al (2013) Protection against malaria by intravenous immunization with a nonreplicating sporozoite vaccine. Science 341: 1359–1365. 10.1126/science.1241800 23929949

[10] White MT, Verity R, Griffin JT, Asante KP, Owusu-Agyei S, et al (2015) Immunogenicity of the RTS,S/AS01 malaria vaccine and implications for duration of vaccine efficacy: secondary analysis of data from a phase 3 randomised controlled trial. Lancet Infect Dis. 10.1016/S1473-3099(15)00239-X PMC4655306 26342424

[11] Matar CG, Anthony NR, O'Flaherty BM, Jacobs NT, Priyamvada L, et al (2015) Gammaherpesvirus Co-infection with Malaria Suppresses Anti-parasitic Humoral Immunity. PLoS Pathog 11: e1004858 10.1371/journal.ppat.1004858 25996913 PMC4440701

[12] von der Weid T, Honarvar N, Langhorne J (1996) Gene-targeted mice lacking B cells are unable to eliminate a blood stage malaria infection. J Immunol 156: 2510–2516. 8786312

[13] Grun JL, Weidanz WP (1981) Immunity to Plasmodium chabaudi adami in the B-cell-deficient mouse. Nature 290: 143–145. 6970898 10.1038/290143a0

[14] van der Heyde HC, Huszar D, Woodhouse C, Manning DD, Weidanz WP (1994) The resolution of acute malaria in a definitive model of B cell deficiency, the JHD mouse. J Immunol 152: 4557–4562. 8157969

[15] Crotty S (2014) T follicular helper cell differentiation, function, and roles in disease. Immunity 41: 529–542. 10.1016/j.immuni.2014.10.004 25367570 PMC4223692

[16] Ueno H, Banchereau J, Vinuesa CG (2015) Pathophysiology of T follicular helper cells in humans and mice. Nat Immunol 16: 142–152. 10.1038/ni.3054 25594465 PMC4459756

[17] Zander RA, Obeng-Adjei N, Guthmiller JJ, Kulu DI, Li J, et al (2015) PD-1 Co-inhibitory and OX40 Co-stimulatory Crosstalk Regulates Helper T Cell Differentiation and Anti-Plasmodium Humoral Immunity. Cell Host Microbe 17: 628–641. 10.1016/j.chom.2015.03.007 25891357 PMC4433434

[18] Choi YS, Kageyama R, Eto D, Escobar TC, Johnston RJ, et al (2011) ICOS receptor instructs T follicular helper cell versus effector cell differentiation via induction of the transcriptional repressor Bcl6. Immunity 34: 932–946. 10.1016/j.immuni.2011.03.023 21636296 PMC3124577

[19] Weber JP, Fuhrmann F, Feist RK, Lahmann A, Al Baz MS, et al (2015) ICOS maintains the T follicular helper cell phenotype by down-regulating Kruppel-like factor 2. J Exp Med 212: 217–233. 10.1084/jem.20141432 25646266 PMC4322049

[20] Choi YS, Gullicksrud JA, Xing S, Zeng Z, Shan Q, et al (2015) LEF-1 and TCF-1 orchestrate TFH differentiation by regulating differentiation circuits upstream of the transcriptional repressor Bcl6. Nat Immunol 16: 980–990. 10.1038/ni.3226 26214741 PMC4545301

[21] Leavenworth JW, Verbinnen B, Yin J, Huang H, Cantor H (2015) A p85alpha-osteopontin axis couples the receptor ICOS to sustained Bcl-6 expression by follicular helper and regulatory T cells. Nat Immunol 16: 96–106. 10.1038/ni.3050 25436971 PMC4405167

[22] Xu H, Li X, Liu D, Li J, Zhang X, et al (2013) Follicular T-helper cell recruitment governed by bystander B cells and ICOS-driven motility. Nature 496: 523–527. 10.1038/nature12058 23619696

[23] Liu D, Xu H, Shih C, Wan Z, Ma X, et al (2015) T-B-cell entanglement and ICOSL-driven feed-forward regulation of germinal centre reaction. Nature 517: 214–218. 10.1038/nature13803 25317561

[24] Akiba H, Takeda K, Kojima Y, Usui Y, Harada N, et al (2005) The role of ICOS in the CXCR5+ follicular B helper T cell maintenance in vivo. J Immunol 175: 2340–2348. 16081804 10.4049/jimmunol.175.4.2340

[25] Wikenheiser DJ, Ghosh D, Kennedy B, Stumhofer JS (2016) The Costimulatory Molecule ICOS Regulates Host Th1 and Follicular Th Cell Differentiation in Response to Plasmodium chabaudi chabaudi AS Infection. J Immunol 196: 778–791. 10.4049/jimmunol.1403206 26667167 PMC4705592

[26] Yu D, Tan AH, Hu X, Athanasopoulos V, Simpson N, et al (2007) Roquin represses autoimmunity by limiting inducible T-cell co-stimulator messenger RNA. Nature 450: 299–303. 10.1038/nature06253 18172933

[27] Pratama A, Ramiscal RR, Silva DG, Das SK, Athanasopoulos V, et al (2013) Roquin-2 shares functions with its paralog Roquin-1 in the repression of mRNAs controlling T follicular helper cells and systemic inflammation. Immunity 38: 669–680. 10.1016/j.immuni.2013.01.011 23583642

[28] Pratama A, Srivastava M, Williams NJ, Papa I, Lee SK, et al (2015) MicroRNA-146a regulates ICOS-ICOSL signalling to limit accumulation of T follicular helper cells and germinal centres. Nat Commun 6: 6436 10.1038/ncomms7436 25743066 PMC4366510

[29] Hall JC, Rosen A (2010) Type I interferons: crucial participants in disease amplification in autoimmunity. Nat Rev Rheumatol 6: 40–49. 10.1038/nrrheum.2009.237 20046205 PMC3622245

[30] Ivashkiv LB, Donlin LT (2014) Regulation of type I interferon responses. Nat Rev Immunol 14: 36–49. 10.1038/nri3581 24362405 PMC4084561

[31] Trinchieri G (2010) Type I interferon: friend or foe? J Exp Med 207: 2053–2063. 10.1084/jem.20101664 20837696 PMC2947062

[32] Le Bon A, Schiavoni G, D'Agostino G, Gresser I, Belardelli F, et al (2001) Type i interferons potently enhance humoral immunity and can promote isotype switching by stimulating dendritic cells in vivo. Immunity 14: 461–470. 11336691 10.1016/s1074-7613(01)00126-1

[33] Cucak H, Yrlid U, Reizis B, Kalinke U, Johansson-Lindbom B (2009) Type I interferon signaling in dendritic cells stimulates the development of lymph-node-resident T follicular helper cells. Immunity 31: 491–501. 10.1016/j.immuni.2009.07.005 19733096

[34] Coro ES, Chang WL, Baumgarth N (2006) Type I IFN receptor signals directly stimulate local B cells early following influenza virus infection. J Immunol 176: 4343–4351. 16547272 10.4049/jimmunol.176.7.4343

[35] Jego G, Palucka AK, Blanck JP, Chalouni C, Pascual V, et al (2003) Plasmacytoid dendritic cells induce plasma cell differentiation through type I interferon and interleukin 6. Immunity 19: 225–234. 12932356 10.1016/s1074-7613(03)00208-5

[36] Banchereau J, Pascual V (2006) Type I interferon in systemic lupus erythematosus and other autoimmune diseases. Immunity 25: 383–392. 10.1016/j.immuni.2006.08.010 16979570

[37] Nakayamada S, Poholek AC, Lu KT, Takahashi H, Kato M, et al (2014) Type I IFN induces binding of STAT1 to Bcl6: divergent roles of STAT family transcription factors in the T follicular helper cell genetic program. J Immunol 192: 2156–2166. 10.4049/jimmunol.1300675 24489092 PMC3967131

[38] Ball EA, Sambo MR, Martins M, Trovoada MJ, Benchimol C, et al (2013) IFNAR1 controls progression to cerebral malaria in children and CD8+ T cell brain pathology in Plasmodium berghei-infected mice. J Immunol 190: 5118–5127. 10.4049/jimmunol.1300114 23585679

[39] Rocha BC, Marques PE, Leoratti FM, Junqueira C, Pereira DB, et al (2015) Type I Interferon Transcriptional Signature in Neutrophils and Low-Density Granulocytes Are Associated with Tissue Damage in Malaria. Cell Rep 13: 2829–2841. 10.1016/j.celrep.2015.11.055 26711347 PMC4698035

[40] Sharma S, DeOliveira RB, Kalantari P, Parroche P, Goutagny N, et al (2011) Innate immune recognition of an AT-rich stem-loop DNA motif in the Plasmodium falciparum genome. Immunity 35: 194–207. 10.1016/j.immuni.2011.05.016 21820332 PMC3162998

[41] Montes de Oca Marcela K R, Rivera Fabian de Labastida, Amante Fiona H., Sheel Meru, et al (2016) Type I interferons regulate immune responses in humans with blood-stage Plasmodium falciparum infection. Cell reports In press. 10.1016/j.celrep.2016.09.015 PMC5082731 27705789

[42] Haque A, Best SE, Montes de Oca M, James KR, Ammerdorffer A, et al (2014) Type I IFN signaling in CD8- DCs impairs Th1-dependent malaria immunity. J Clin Invest 124: 2483–2496. 10.1172/JCI70698 24789914 PMC4038565

[43] Edwards CL, Best SE, Gun SY, Claser C, James KR, et al (2015) Spatiotemporal requirements for IRF7 in mediating type I IFN-dependent susceptibility to blood-stage Plasmodium infection. Eur J Immunol 45: 130–141. 10.1002/eji.201444824 25319247

[44] Ray JP, Marshall HD, Laidlaw BJ, Staron MM, Kaech SM, et al (2014) Transcription factor STAT3 and type I interferons are corepressive insulators for differentiation of follicular helper and T helper 1 cells. Immunity 40: 367–377. 10.1016/j.immuni.2014.02.005 24631156 PMC3992517

[45] Suss G, Eichmann K, Kury E, Linke A, Langhorne J (1988) Roles of CD4- and CD8-bearing T lymphocytes in the immune response to the erythrocytic stages of Plasmodium chabaudi. Infect Immun 56: 3081–3088. 2903123 10.1128/iai.56.12.3081-3088.1988 PMC259705

[46] Yong PF, Salzer U, Grimbacher B (2009) The role of costimulation in antibody deficiencies: ICOS and common variable immunodeficiency. Immunol Rev 229: 101–113. 10.1111/j.1600-065X.2009.00764.x 19426217

[47] McAdam AJ, Greenwald RJ, Levin MA, Chernova T, Malenkovich N, et al (2001) ICOS is critical for CD40-mediated antibody class switching. Nature 409: 102–105. 10.1038/35051107 11343122

[48] Baumjohann D, Preite S, Reboldi A, Ronchi F, Ansel KM, et al (2013) Persistent antigen and germinal center B cells sustain T follicular helper cell responses and phenotype. Immunity 38: 596–605. 10.1016/j.immuni.2012.11.020 23499493

[49] Haque A, Best SE, Ammerdorffer A, Desbarrieres L, de Oca MM, et al (2011) Type I interferons suppress CD4(+) T-cell-dependent parasite control during blood-stage Plasmodium infection. Eur J Immunol 41: 2688–2698. 10.1002/eji.201141539 21674481

[50] Obeng-Adjei N, Portugal S, Tran TM, Yazew TB, Skinner J, et al (2015) Circulating Th1-Cell-type Tfh Cells that Exhibit Impaired B Cell Help Are Preferentially Activated during Acute Malaria in Children. Cell Rep 13: 425–439. 10.1016/j.celrep.2015.09.004 26440897 PMC4607674

[51] Ryg-Cornejo V, Ioannidis LJ, Ly A, Chiu CY, Tellier J, et al (2016) Severe Malaria Infections Impair Germinal Center Responses by Inhibiting T Follicular Helper Cell Differentiation. Cell Rep 14: 68–81. 10.1016/j.celrep.2015.12.006 26725120

[52] Aucan C, Walley AJ, Hennig BJ, Fitness J, Frodsham A, et al (2003) Interferon-alpha receptor-1 (IFNAR1) variants are associated with protection against cerebral malaria in the Gambia. Genes Immun 4: 275–282. 10.1038/sj.gene.6363962 12761564

[53] Pichyangkul S, Yongvanitchit K, Kum-arb U, Hemmi H, Akira S, et al (2004) Malaria blood stage parasites activate human plasmacytoid dendritic cells and murine dendritic cells through a Toll-like receptor 9-dependent pathway. J Immunol 172: 4926–4933. 15067072 10.4049/jimmunol.172.8.4926

[54] Kim CC, Nelson CS, Wilson EB, Hou B, DeFranco AL, et al (2012) Splenic red pulp macrophages produce type I interferons as early sentinels of malaria infection but are dispensable for control. PLoS One 7: e48126 10.1371/journal.pone.0048126 23144737 PMC3483282

[55] Voisine C, Mastelic B, Sponaas AM, Langhorne J (2010) Classical CD11c+ dendritic cells, not plasmacytoid dendritic cells, induce T cell responses to Plasmodium chabaudi malaria. Int J Parasitol 40: 711–719. 10.1016/j.ijpara.2009.11.005 19968996

[56] Findlay EG, Greig R, Stumhofer JS, Hafalla JC, de Souza JB, et al (2010) Essential role for IL-27 receptor signaling in prevention of Th1-mediated immunopathology during malaria infection. J Immunol 185: 2482–2492. 10.4049/jimmunol.0904019 20631310

[57] Li C, Corraliza I, Langhorne J (1999) A defect in interleukin-10 leads to enhanced malarial disease in Plasmodium chabaudi chabaudi infection in mice. Infect Immun 67: 4435–4442. 10456884 10.1128/iai.67.9.4435-4442.1999 PMC96762

[58] Choi YS, Eto D, Yang JA, Lao C, Crotty S (2013) Cutting edge: STAT1 is required for IL-6-mediated Bcl6 induction for early follicular helper cell differentiation. J Immunol 190: 3049–3053. 10.4049/jimmunol.1203032 23447690 PMC3626564

[59] Dienz O, Eaton SM, Bond JP, Neveu W, Moquin D, et al (2009) The induction of antibody production by IL-6 is indirectly mediated by IL-21 produced by CD4+ T cells. J Exp Med 206: 69–78. 10.1084/jem.20081571 19139170 PMC2626667

[60] Harker JA, Dolgoter A, Zuniga EI (2013) Cell-intrinsic IL-27 and gp130 cytokine receptor signaling regulates virus-specific CD4(+) T cell responses and viral control during chronic infection. Immunity 39: 548–559. 10.1016/j.immuni.2013.08.010 23993651 PMC4701058

[61] Harker JA, Lewis GM, Mack L, Zuniga EI (2011) Late interleukin-6 escalates T follicular helper cell responses and controls a chronic viral infection. Science 334: 825–829. 10.1126/science.1208421 21960530 PMC3388900

[62] Shin C, Han JA, Koh H, Choi B, Cho Y, et al (2015) CD8alpha(-) Dendritic Cells Induce Antigen-Specific T Follicular Helper Cells Generating Efficient Humoral Immune Responses. Cell Rep 11: 1929–1940. 10.1016/j.celrep.2015.05.042 26095362

[63] Li J, Lu E, Yi T, Cyster JG (2016) EBI2 augments Tfh cell fate by promoting interaction with IL-2-quenching dendritic cells. Nature 533: 110–114. 10.1038/nature17947 27147029 PMC4883664

[64] Karunarathne DS, Horne-Debets JM, Huang JX, Faleiro R, Leow CY, et al (2016) Programmed Death-1 Ligand 2-Mediated Regulation of the PD-L1 to PD-1 Axis Is Essential for Establishing CD4(+) T Cell Immunity. Immunity 45: 333–345. 10.1016/j.immuni.2016.07.017 27533014

[65] Warnatz K, Bossaller L, Salzer U, Skrabl-Baumgartner A, Schwinger W, et al (2006) Human ICOS deficiency abrogates the germinal center reaction and provides a monogenic model for common variable immunodeficiency. Blood 107: 3045–3052. 10.1182/blood-2005-07-2955 16384931

[66] Khoury DS, Cromer D, Best SE, James KR, Kim PS, et al (2014) Effect of mature blood-stage Plasmodium parasite sequestration on pathogen biomass in mathematical and in vivo models of malaria. Infect Immun 82: 212–220. 10.1128/IAI.00705-13 24144725 PMC3911827

[67] Amante FH, Good MF (1997) Prolonged Th1-like response generated by a Plasmodium yoelii-specific T cell clone allows complete clearance of infection in reconstituted mice. Parasite Immunol 19: 111–126. 9106817 10.1046/j.1365-3024.1997.d01-187.x

[68] Su Z, Tam MF, Jankovic D, Stevenson MM (2003) Vaccination with novel immunostimulatory adjuvants against blood-stage malaria in mice. Infect Immun 71: 5178–5187. 10.1128/IAI.71.9.5178-5187.2003 12933862 PMC187300

[69] Amante FH, Stanley AC, Randall LM, Zhou Y, Haque A, et al (2007) A role for natural regulatory T cells in the pathogenesis of experimental cerebral malaria. Am J Pathol 171: 548–559. 10.2353/ajpath.2007.061033 17600128 PMC1934517

[70] Haque A, Best SE, Amante FH, Mustafah S, Desbarrieres L, et al (2010) CD4+ natural regulatory T cells prevent experimental cerebral malaria via CTLA-4 when expanded in vivo. PLoS Pathog 6: e1001221 10.1371/journal.ppat.1001221 21170302 PMC3000360

[71] Beattie L, Peltan A, Maroof A, Kirby A, Brown N, et al (2010) Dynamic imaging of experimental Leishmania donovani-induced hepatic granulomas detects Kupffer cell-restricted antigen presentation to antigen-specific CD8 T cells. PLoS Pathog 6: e1000805 10.1371/journal.ppat.1000805 20300603 PMC2837408

[72] Veiga-Fernandes H, Coles MC, Foster KE, Patel A, Williams A, et al (2007) Tyrosine kinase receptor RET is a key regulator of Peyer's patch organogenesis. Nature 446: 547–551. 10.1038/nature05597 17322904

