# Supplementary figures and images for: IFNAR1-Signalling Obstructs ICOS-mediated Humoral Immunity during Non-lethal Blood-Stage Plasmodium Infection
Source: PLoS Pathog. 2016 Nov 3;12(11):e1005999. doi: 10.1371/journal.ppat.1005999 (PMC5094753; doi:10.1371/journal.ppat.1005999)

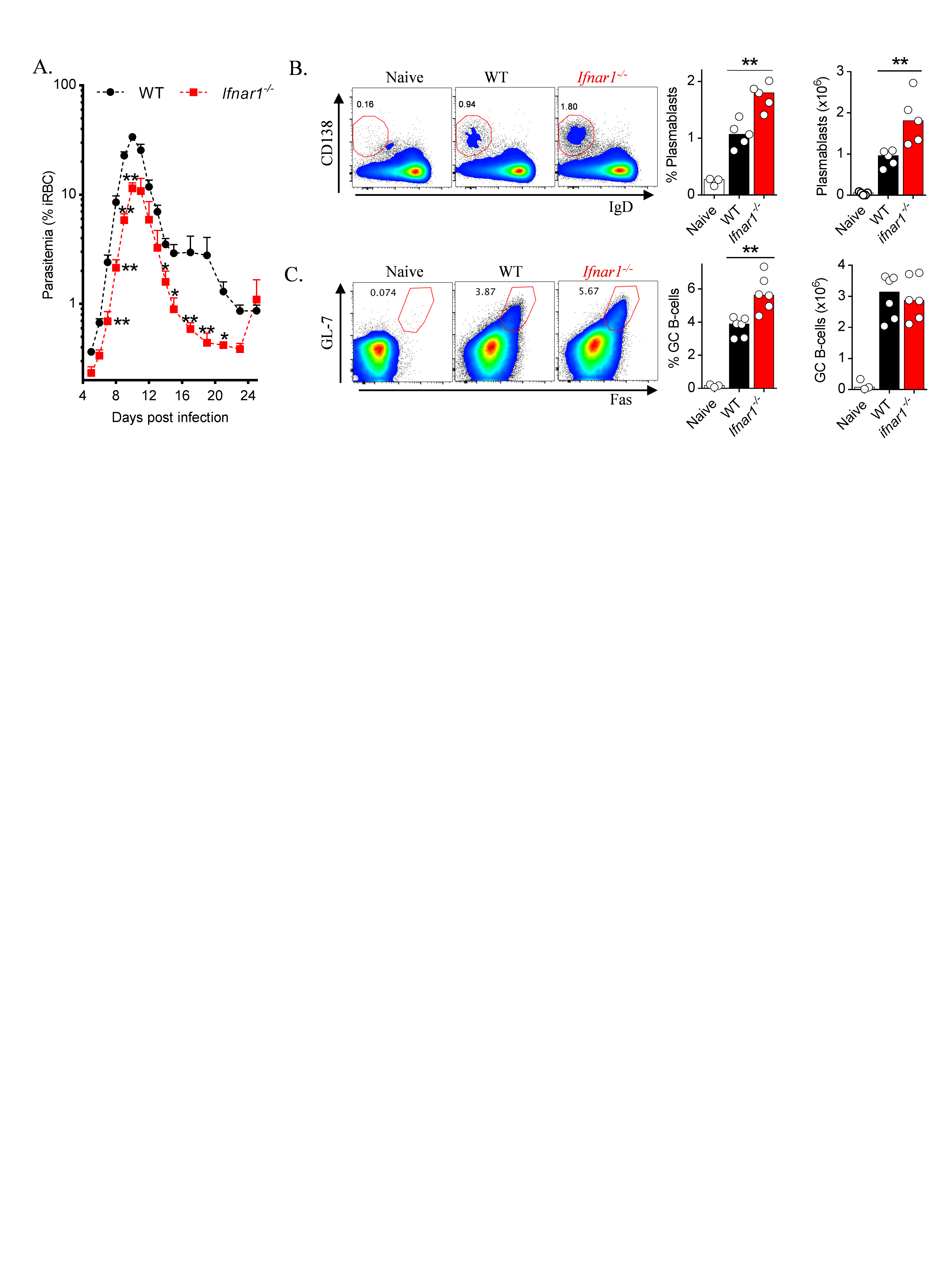

Supplement: S1 Fig — WT and Ifnar1 -/- mice (n = 5–9) were infected with PcAS. (A) A time-course analysis of parasitemia in WT (n = 9/group) and Ifnar1 -/-(n = 6/group) mice Data representative of three independent experiments. Statistics: Mann-Whitney U test, **P<0.01, *P<0.05. (B&C) Representative FACS plots (gated on B220+ CD19+ live singlets), proportions and absolute numbers of (B) splenic plasmablasts (B220+CD19+IgDloCD138hi) in naïve and infected mice, 6 days p.i., and (C) emerging splenic GC B-cells (B220+CD19+GL7+Fas+) in naïve and infected mice, 8 days p.i. Data in B&C representative of three independent experiments. Statistics: Mann-Whitney U test, **P<0.01. (TIF) [file ppat.1005999.s001.tif]

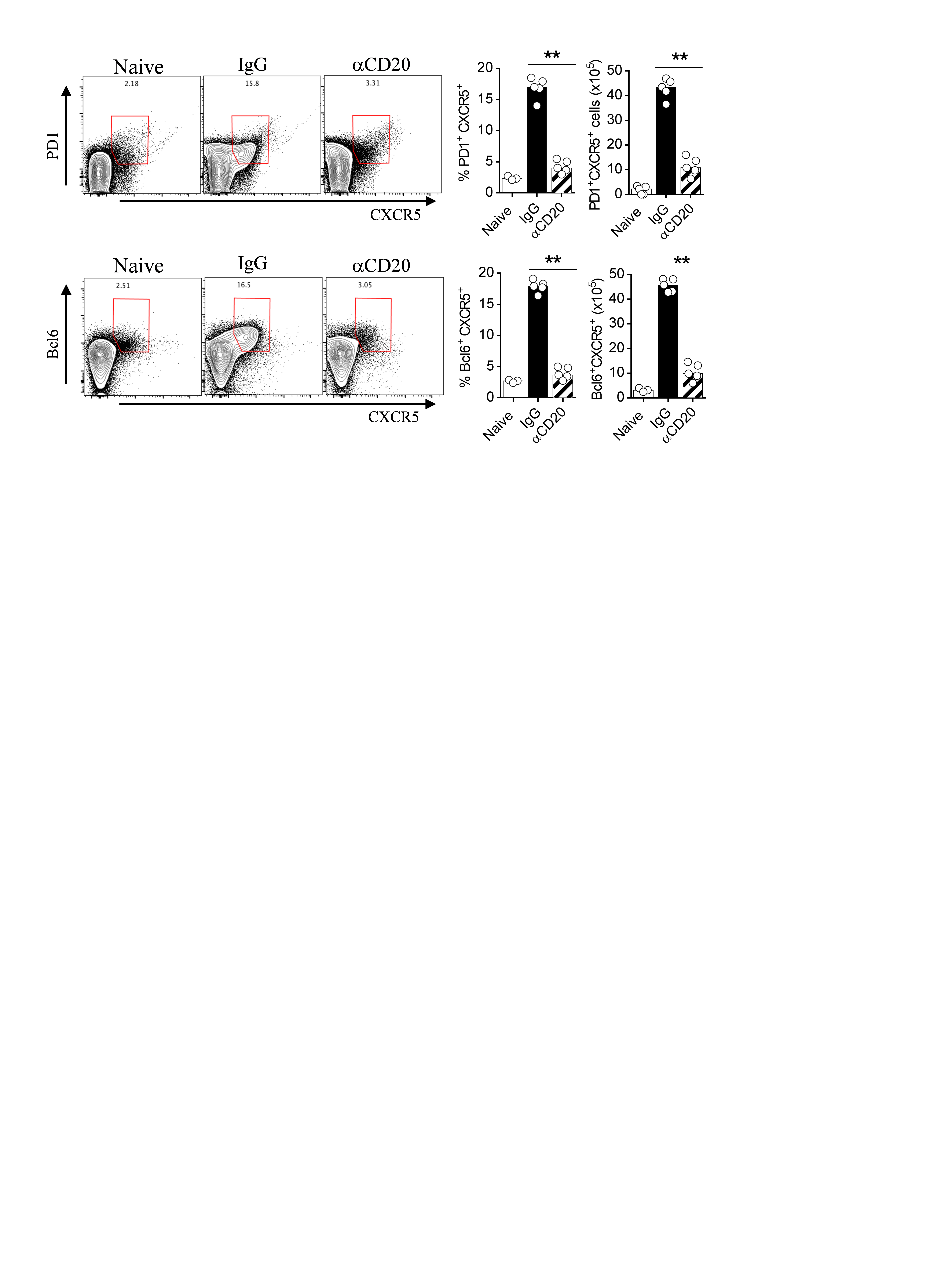

Supplement: S2 Fig — WT mice (n = 5/group) were pre-treated with anti-CD20 depleting monoclonal antibody (α-CD20) or control-IgG before infection with PcAS. Representative FACS plots (gated on CD4+ TCRβ+ live singlets), proportions and absolute numbers of splenic CD4+ T cells co-expressing PD1/CXCR5 and Bcl-6/CXCR5, 7 days p.i. Data representative of two independent experiments. Mann-Whitney U test, **P<0.01. (TIF) [file ppat.1005999.s002.tif]

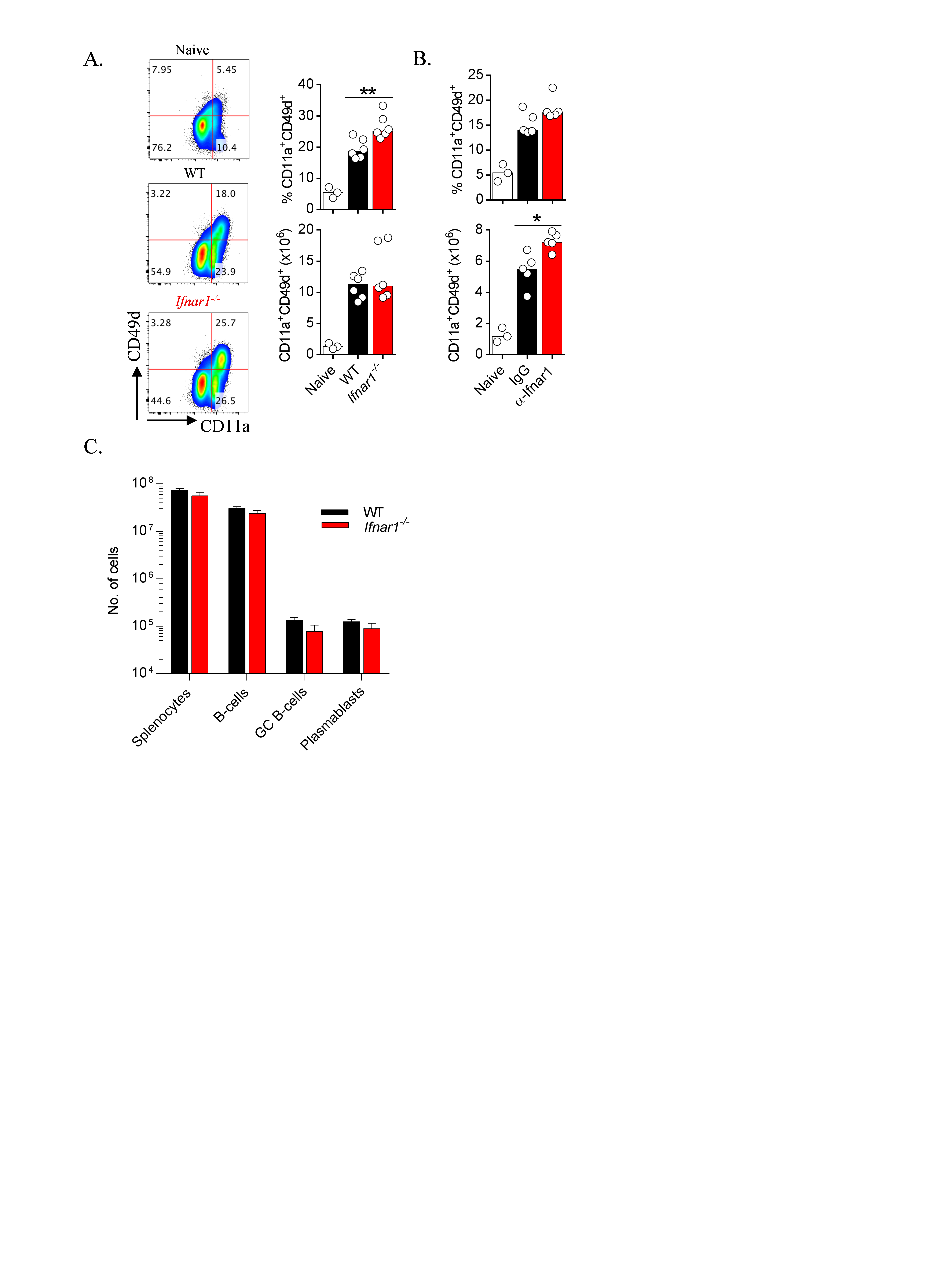

Supplement: S3 Fig — (A). Representative FACS plots, proportions and numbers of splenic activated CD4+ T-cells (CD4+TCRβ+CD11a+CD49d+ live singlets) in WT and Ifnar1 -/- mice (n = 6) 8 days p.i. with PcAS. (B) Proportions and numbers of splenic activated CD4+ T-cells on day 6 p.i. with Py17XNL in WT mice (n = 5) treated with α-Ifnar1 or control IgG. (C) Total numbers of splenocytes, B-cells, GC B-cells and plasmablasts in un-infected WT and Ifnar1 -/- mice (n = 5). Experiments performed once. Statistics: Mann-Whitney U test, *P<0.05; **P<0.01. (TIF) [file ppat.1005999.s003.tif]

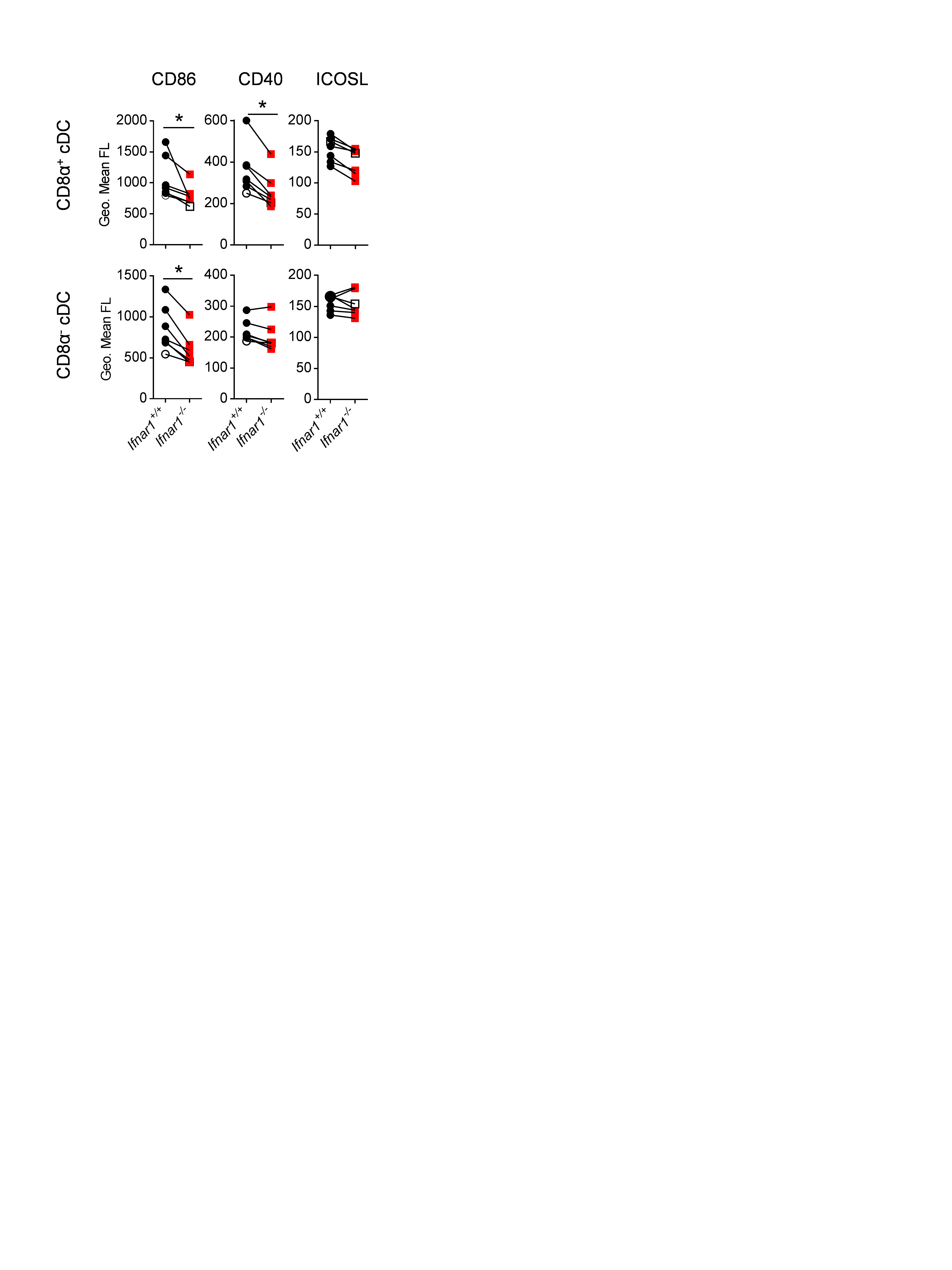

Supplement: S4 Fig — 50:50 WT (Ifnar1 +/+ CD45.1): Ifnar1 -/- (CD45.2) mixed bone marrow chimeras (n = 6) were infected with PcAS. Graphs show paired analysis between splenic WT and Ifnar1 -/- cDC subsets in individual mice for cell-surface expression of CD86, CD40 and ICOS-L, on CD8α+ (TCRβ- B220- CD11chi MHC-IIhi CD8α+) and CD8α- (TCRβ-B220-CD11chiMHC-IIhiSirpα+CD8-) cDCs, 2 days p.i. (experiment performed once). White symbols denote un-infected control. Statistics: Wilcoxon test, *P<0.05. (TIF) [file ppat.1005999.s004.tif]

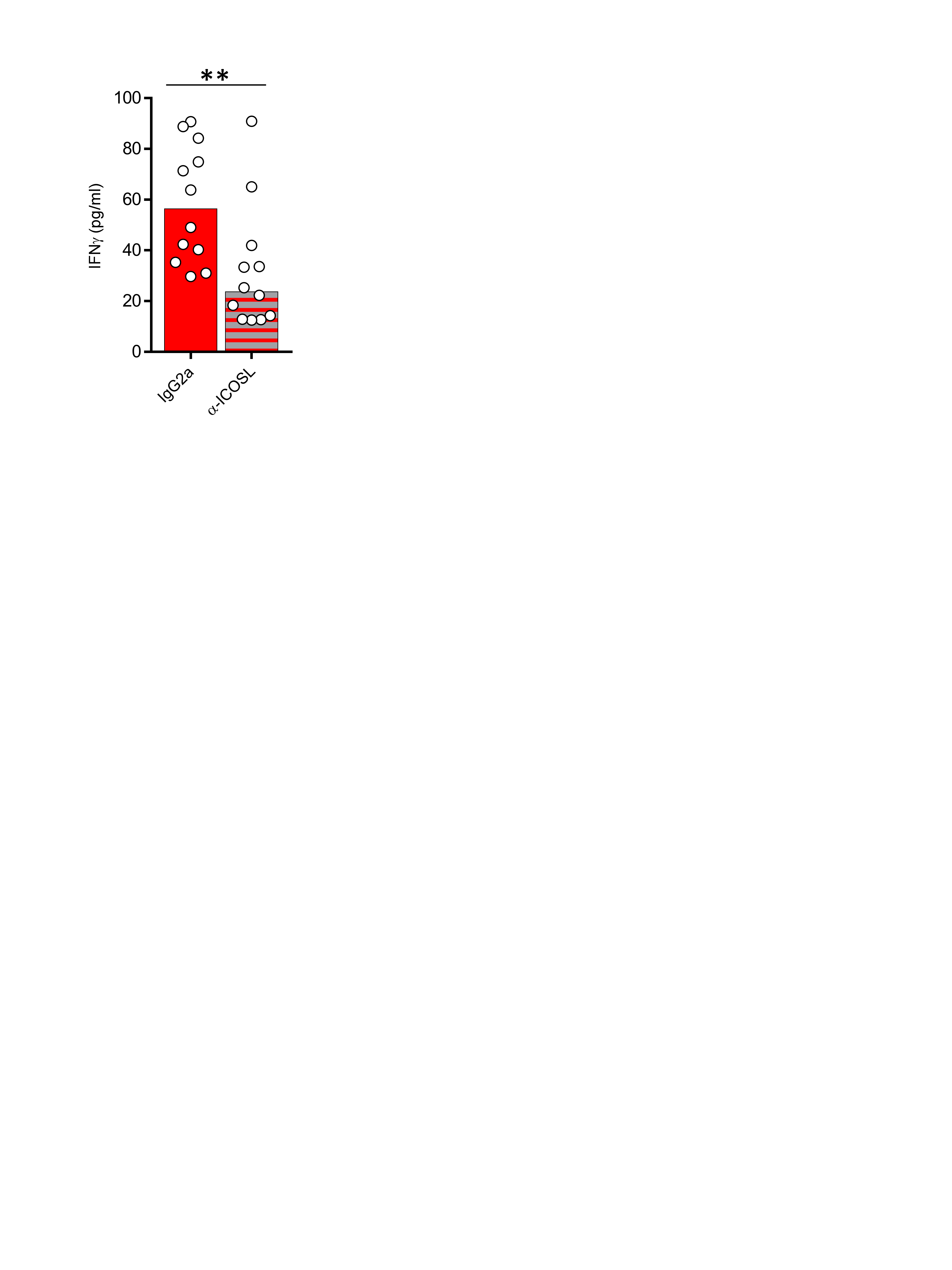

Supplement: S5 Fig — Ifnar1 -/- mice were infected with PcAS, treated with α-ICOSL (100μg) or control IgG2a and assessed for IFNγ levels in serum on day 8 p.i. Data is pooled from two independent experiments showing similar results (n = 6/ experiment). Statistics: Mann-Whitney U test, **P<0.01. (TIF) [file ppat.1005999.s005.tif]
